# Refining the Design of Diblock Elastin-Like Polypeptides for Self-Assembly into Nanoparticles

**DOI:** 10.3390/polym13091470

**Published:** 2021-05-01

**Authors:** Michèle Dai, Evangelos Georgilis, Guillaume Goudounet, Bertrand Garbay, Jan Pille, Jan C. M. van Hest, Xavier Schultze, Elisabeth Garanger, Sébastien Lecommandoux

**Affiliations:** 1 University Bordeaux, CNRS, Bordeaux INP, LCPO, UMR 5629, 33600 Pessac, France; dai.michele.sl@gmail.com (M.D.); evangelosmgeorgilis@gmail.com (E.G.); guillaume.goudounet@orange.fr (G.G.); Bertrand.Garbay@enscbp.fr (B.G.); 2 L’Oréal Recherche Avancée, 1 Avenue Eugène Schueller, 93600 Aulnay-sous-Bois, France; xavier.schultze@rd.loreal.com; 3 Current affiliation E.G. (Evangelos Georgilis): CIC nanoGUNE (BRTA), Tolosa Hiribidea 76, 20018 Donostia-San Sebastián, Spain; 4 Bio-organic Chemistry Lab, Eindhoven University of Technology, P.O. Box 513 (STO 3.31), 5600 MB Eindhoven, The Netherlands; mail@janpille.org (J.P.); J.C.M.v.Hest@tue.nl (J.C.M.v.H.)

**Keywords:** elastin-like polypeptides, thermoresponsiveness, self-assembly, nanoparticles, methionine oxidation

## Abstract

Diblock copolymers based-on elastin-like polypeptide (ELP) have the potential to undergo specific phase transitions when thermally stimulated. This ability is especially suitable to form carriers, micellar structures for instance, for delivering active cargo molecules. Here, we report the design and study of an ELP diblock library based on ELP-[M_1_V_3_-*i*]-[I-*j*]. First, ELP-[M_1_V_3_-*i*]-[I-*j*] (*i* = *20*, *40*, *60*; *j* = *20*, *90*) that showed a similar self-assembly propensity (unimer-to-aggregate transition) as their related monoblocks ELP-[M_1_V_3_-*i*] and ELP-[I-*j*]. By selectively oxidizing methionines of ELP-[M_1_V_3_-*i*] within the different diblocks structures, we have been able to access a thermal phase transition with three distinct regimes (unimers, micelles, aggregates) characteristic of well-defined ELP diblocks.

## 1. Introduction

Thermoresponsive polymers are attractive smart materials, in particular for applications in biomedical sciences (i.e., biomedicine). They exhibit a reversible phase transition behavior in aqueous medium upon temperature changes and come in two categories, namely, those that become soluble above an upper critical solution temperature (UCST) and those that conversely aggregate above a lower critical solution temperature (LCST) [1,2,3,4]. The thermal phase transition of these polymers in aqueous solutions has been and is still extensively studied, and significant efforts in terms of design and synthesis are provided to fine tune their physico-chemical characteristics in relevance with biomedical applications, namely in temperature ranges close to physiological body temperature. 

To date, there is a limited number of commercial products based on thermoresponsive polymers which mainly find uses in the control of blood flow and release of therapeutics [3,4,5,6]. However, a considerable amount of work is being invested in order to develop novel smart drug delivery systems based on thermoresponsive polymers [7]. UCST-polymers in aqueous medium (e.g., poly(*N*-acryloylglycinamide), poly(acrylamide-*co*-acrylonitrile), polybetaine) are much less reported than LCST-polymers but have aroused a great interest in the last decade [2,3,8]. Polymers exhibiting a UCST have been studied for the development of drug delivery systems to release therapeutics under hyperthermic conditions [9]. In contrast, LCST-polymers come in a wider variety and comprise synthetic polymers such as poly(*N*-isopropylacrylamide) (PNIPAM) [10], the most extensively studied LCST-polymer, but also poly(oligo ethylene glycol methacrylate) (P(OEGMA)) [11], poly(2-oxazoline) (POx) [12], polypeptoids [13], poly(N-vinylcaprolactam) [14] and certain block copolymers [15,16]. 

**Figure 1:**
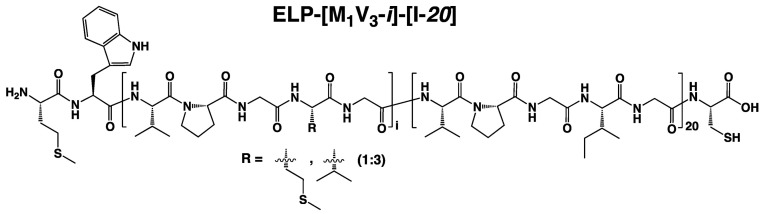
Schematic representation and chemical structure of the ELP-[M_1_V_3_-*i*]-[I-*20*] used in this work.

Apart from these, another category of macromolecules showing LCST behavior has gained significant attention in the past two decades, namely elastin-like polypeptides (ELPs) [17]. The most commonly studied ELPs are composed of repeating units of -VPGXG- pentapeptides where X is a guest residue that can be any amino acid except proline (Figure 1) [18]. The repeated sequences have been rationally chosen from the large domains of tropoelastin, the natural precursor of elastin. The repeat of these five pentapeptides promotes chains rigidity through proline frequency and flexibility through glycine motifs. Thus, the combined proline-glycine contributions keep the protein in a transient structure, both solvated and aggregated states. While the guest residue X allows to variate the charge and hydrophobicity of the ELP and then, tune its chain conformation, like the n repeats [19]. However, if the guest residue X is a proline, it would disrupt the ELP chain conformation.

The first pioneering works on the thermal phase behavior of ELPs were conducted by Dan W. Urry and his colleagues, who showed that synthetic polypeptides based on repeated -VPGVG- sequences coacervate above a specific temperature in a similar manner as α-elastin [20,21]. Urry also indicated how the hydrophobicity of the amino acid occupying the guest residue position affects this specific temperature (noted T_t_) corresponding to the cloud point temperature of the solution, and established a hydrophobicity scale to predict the effect of a specific residue on the thermal responsiveness of an ELP [22,23]. 

The chemical synthesis of ELPs by polycondensation was later replaced by recombinant expression in bacteria, as a method providing greater control over the sequence and molecular weight of the polypeptide as well as a perfect batch-to-batch reproducibility [24]. The construction of ELP genes for heterologous expression became easier thanks to the progress in the cloning of repetitive genes, especially with the method of recursive directional ligation (RDL) developed by Chilkoti et al. [25,26]. RDL and its variants enabled the construction of different ELP architectures such as block copolymers. Hence, it became possible to explore various libraries of ELPs in order to understand and predict their thermal behavior as a function of their molecular weight and concentration [27]. Further investigations showed the potential applications of ELPs as bioconjugates that thermally target and/or enhance the pharmacokinetic properties of active pharmaceutical ingredients [28], depots for drug delivery [29,30], and micelles with targeting properties [31], to name a few.

ELP-based micelles can be fabricated either from ELP block copolymers or hybrids with other macromolecules [32,33]. The first category mainly includes diblock ELPs where each block contains guest residues of different hydrophilicity [34,35,36,37] exhibiting two distinct thermal transitions as a result of their transition temperature difference as well as the length per block [38,39,40]. The first transition corresponds to the collapse of the most hydrophobic ELP block at a temperature defined as the critical micelle temperature (CMT). The second transition corresponds to the collapse of the most hydrophilic block above the bulk transition temperature (T_t,bulk_), resulting in the formation of macroscopic aggregates. Between the CMT and T_t,bulk_ is the regime where diblock ELPs self-assemble into micelles [39]. MacKay et al. developed a quantitative mathematical model which relates the CMT and T_t,bulk_ to the phase behavior of the monoblock counterparts, thus expanding the previous empirical model of Chilkoti to diblock systems [38].

In the majority of cases, the hydrophilic segment of diblock ELPs contains amino acids that are not amenable to chemical modifications (e.g., alanine or serine). However, residues such as methionine can be introduced to ELPs to tune their properties by applying chemical modifications [41,42]. This is why this work focuses on the thermal behavior of diblock ELPs comprised of a *C*-terminal hydrophobic segment with isoleucine as a guest residue and a less hydrophobic segment containing methionine as a modifiable guest residue (Figure 1). 

It was demonstrated that chemoselective modifications of the methionine thioether could serve as a useful tool to tune the thermal behavior of these diblocks and lead to their thermal transition into micelles depending on block length.

## 2. Materials and Methods

The ELP nomenclature provided in this work is the one proposed by Chilkoti, i.e., (A_x_B_y_C_z_-*n*), where A, B and C denote the guest residues, and x:y:z their ratio within the whole ELP, and *n* the total number of pentapeptide repeats [25].

### 2.1. Materials

Lysogeny Broth (LB) medium and hydrogen peroxide (H_2_O_2_) solution 30% *w*/*w* in water were purchased from Sigma-Aldrich (Saint Quentin Fallavier, France). The phosphorylated oligonucleotide pCGTAGG was obtained from Eurogentec (Seraing, Belgium). Yeast extract was purchased from Biokar Diagnostics (FR). Ampicillin was obtained from Euromedex (Souffelweyersheim, France). Phosphate Buffer Saline (PBS) 10× purchased also from Euromedex was diluted to 1× for ELP solutions. Glycerol and isopropyl β-D-1-thiogalactopyranoside (IPTG) were obtained from Fisher Scientific (Illkirch Graffenstaden, France). Tris(2-carboxyethyl)phosphine hydrochloride (TCEP∙HCl) and *N*-ethylmaleimide (NEM) were obtained from TCI Chemicals (Belgium). *N*-ethyldiisopropylamine (DIPEA) and dimethylformamide (DMF) were purchased from Alfa Aesar (Schiltigheim, France). Ultrapure (UP) water (18 MΩ-cm) was obtained by using a Millipore Milli-Q Biocel A10 purification unit. 

### 2.2. Production of ELP-[M_1_V_3_-i] (i = 20, 40, 60, 80, 100) Monoblocks

These five ELPs were produced following a previously reported procedure [41].

### 2.3. Production and Post-Modification of ELP-[I-j] (j = 20, 40, 60) Monoblocks

ELP-[I-*j*] (*j* = *20*, *40*, *60*) were produced following a previously reported procedure [43]. The thiol side chain of the *C*-terminal residue was capped with *N-*ethylmaleimide. Briefly, in a 10 mL vial, ELP (0.003 mmol, 1 equiv.) and TCEP∙HCl (4.22 mg, 0.015 mmol, 5 equiv.) were dissolved in 2.5 mL anhydrous DMF. The solution was flushed for 30 min with nitrogen. 7.69 µL DIPEA (0.044 mmol, 15 equiv.) and 2.5 mL of a solution containing NEM (3.68 mg, 0.03 mmol, 10 equiv.) in anhydrous DMF were added. After 24 h, the mixture was diluted in UP water, dialyzed against UP water using 3 kDa-cut off dialysis tubing, and freeze-dried to yield a white fluffy compound.

### 2.4. Production and Post-Modification of ELP-[M_1_V_3_-i]-[I-20] (i = 20, 40, 60)

#### 2.4.1. Construction of the Expression Vector ELP-[M_1_V_3_-*i*]-[I-*20*] (*i* = *20*, *40*, *60*)

The diblocks were produced based on a previous protocol [44]. Briefly, by using a variation of the recursive directional ligation method, the sequences coding for ELP-[M_1_V_3_-*i*]-[I-*20*] (*i* = *20*, *40*, *60*) were obtained. The ELP-[M_1_V_3_-*20*] sequence was extracted from the pUC19-ELP-[M_1_V_3_-*20*] by a double digestion with *BsmFI* and *BtgZI*, and was ligated by using the Quick ligation^TM^ kit into the *BsmFI*-digested and dephosphorylated pUC19-ELP-[I-*20*] plasmid. A ratio 5:1 insert:vector (ELP-[M_1_V_3_-*20*]:ELP-[I-*20*]) was used for the ligation to optimize insertion of multiple ELP-[M_1_V_3_-*20*] fragments, and thus to obtain ELP-[M_1_V_3_-*i*]-[I-*20*] (*i* = *20*, *40*, *60*). After transformation of NEB 5α-F’Iq *E. coli* competent cells, positive clones were selected by colony PCR with OneTaq® hot start DNA polymerase and verified by DNA sequencing (GATC biotech AG, Germany). The different ELP diblock sequences were then extracted from pUC19-ELP-[M_1_V_3_-*i*]-[I-*20*] (*i* = *20*, *40*, *60*) by a double digestion *NdeI* and *BamHI*, and ligated with the Quick ligation™ kit into similarly digested and dephosphorylated pET-44a(+) plasmid. The different ligation products were then used to transform NEB5α-F’Iq *E. coli* competent cells. Positive clones were identified by PCR screening. The plasmids were then purified, sequenced, and used to transform BLR(DE3)-competent cells for production. The sequences of the ELP genes and of the corresponding proteins are shown in supporting information (Figure S1).

#### 2.4.2. Production

For each ELP, a single bacterial colony was selected and cultured overnight at 37 °C in a rotary shaker at 200 rpm in 50 mL of LB-rich medium (1% bacto tryptone, 0.5% NaCl, 1% yeast extract) supplemented with 2.5 g/L glycerol and ampicillin (100 µg/mL). The seed culture was inoculated into 1 L LB-rich medium supplemented with glycerol (2.5 g/L) and ampicillin (100 µg/mL), and bacteria were cultivated at 37 °C in a 5 L flask. When the OD_600nm_ reached a value close to 0.8, IPTG was added to a final concentration of 0.5 mM and the temperature of cultivation was decreased to 25 °C. After 21 h of IPTG induction, cells were harvested for purification. Samples were taken at different times of the culture process and their protein content was analyzed by SDS-PAGE (Figure S2).

#### 2.4.3. Purification

After 21 h of IPTG-induction, the culture was harvested by centrifugation at 7500× *g* and 4 °C for 15 min, and the cell pellet was resuspended with ultrapure water to target 10 mL/g wet weight. The sample was incubated overnight at −80 °C and slowly defrosted by incubation at 4 °C. Cell lysis was completed by sonication at 15 °C with sequential 4 s-pulses at 125 W separated by 8 s-resting time periods for a total duration of 45 min. Insoluble debris were removed by centrifugation at 11,000× *g* and 4 °C for 20 min. The cleared lysate was thereafter subjected to three successive rounds of inverse transition cycling (ITC) [45]. The ELP was precipitated by increasing temperature at 30 °C during 30 min and retrieved by centrifugation at 7500× *g* and 30 °C for 15 min (“Hot spin”). After elimination of the supernatant containing the soluble proteins, the pellet was resuspended in cold ultrapure water to solubilize the ELP. Insoluble, heat denatured proteins from *E. coli* were eliminated by centrifugation at 7500× *g* and 4 °C for 15 min (“Cold spin”). The ELP-containing supernatant was then subjected to additional ITC rounds. Finally, the purified ELP was extensively dialyzed against ultrapure water at 4 °C using 3 kDa MWCO-dialysis tubing (Spectra Por7), and then freeze-dried. Through this procedure, pure ELP-[M_1_V_3_-*i*]-[I-*20*] (*i* = 20, 40, 60) white fluffy compounds were obtained. Mass spectrometry analysis was performed and experimental masses of 17,385 Da, 25,734 Da and 34,001 Da were found for ELP-[M_1_V_3_-*i*]-[I-*20*] (*i* = 20, 40, 60), respectively (Figure S3). The ^1^H NMR spectra in Figure S4 of each ELP were also recorded in D_2_O and carefully assigned. 

#### 2.4.4. NEM Capping of Cysteine C-Terminal 

The *C*-terminal cysteine residue of each ELP diblock was capped according to the protocol described in 2.3 (mean yield > 60%). The obtained products were characterized through SDS-PAGE and SEC in DMF (Figure S5).

### 2.5. Production and Post-Modification of ELP-[M_1_V_3_-i]-[I-90], (i = 40, 60)

#### 2.5.1. Construction of the Expression Vector ELP-[M_1_V_3_-*i*]-[I-*90*] (*i* = *40*, *60*)

The construction of the DNA sequence coding ELP-[M_1_V_3_-*i*]-[I-*90*] (*i* = *40*, *60*) was based on a different strategy. The ELP-[M_1_V_3_-*i*] blocks were isolated from a pET-44a(+) vector by single digestion with *Bsm*FI, followed by a double digestion with the restriction enzymes *Xba*I and *Btg*ZI. In parallel, a pET-24a vector containing the ELP-[I-*90*] gene (plasmid donated by the group of Jan van Hest, TU/e, the Netherlands) was digested by *Xba*I and *Bse*RI. The ends generated by the *Bse*RI and *Btg*ZI digestion being non-compatible, the ligation between the ELP-[M_1_V_3_-*i*] inserts and the ELP-[I-*90*] requires the addition of a 5′ phosphorylated oligonucleotide, pCGTAGG to ‘bridge” the two fragments. The ligation reaction was done at a molar ratio of 1:6:60 for, respectively the vector, the insert gene, and the bridging: oligonucleotide. A schematic representation of the cloning strategy to obtain ELP-[M_1_V_3_-*i*]-[I-*90*] in pET-24a can be found in the supporting information (Scheme S1). After cloning, plasmids were purified, verified by digestion using *Bam*HI and *Xba*I (Figure S6), and sequenced. The plasmids containing the diblock ELP genes were then used to transform BL21 Star™ (DE3) competent cells for ELP production.

#### 2.5.2. Production of ELP-[M_1_V_3_-*i*]-[I-*90*], (*i* = *40*, *60*)

The production protocol for these diblocks was as described in the Section 2.4.2, with the difference that the expression was carried out at 28 °C for 72 h. SDS-PAGE of the productions are available in Figure S7.

#### 2.5.3. Purification 

The protocol followed is similar to the one described in 2.4.3, with the difference that ammonium sulfate was used for the precipitation of the ELPs during the ITC cycles and DMSO was employed to aid their resolubilization. In particular, the ELP-containing supernatant was supplemented with 10% *v*/*v* saturated ammonium sulfate solution to aggregate the ELPs at room temperature. The first hot spin was conducted by centrifugation at 10,000 rpm, 15 min, 25 °C, and the ELP pellets were resolubilized in DMSO after a freeze–thaw cycle. The mixture was diluted with cold 15 mM phosphate 15 mM NaCl buffer pH 7.4 (final content of DMSO 10% *v*/*v*) before proceeding to the first cold spin at 15,000 rpm, 10 min, 4 °C. The ITC cycles were repeated another 3 times without the use of DMSO. For each cold spin, the volume of buffer to resolubilize the ELPs was reduced to half, and thus for every hot spin the volume of ammonium sulfate was also reduced to half. The supernatant isolated after cold spin 4 was desalted on a HiPrep 26/10 (GE Healthcare Life Sciences) with an AKTA Explorer 10 (GE Healthcare Life Sciences) at 1 mL/min ultrapure water. 

### 2.6. Chemical Oxidation of Diblock ELPs

ELP (1 equiv.) was dissolved in ultrapure water (10 mg/mL) containing 10 mM Tris pH 7.8. Then, 1.8% *v*/*v* of H_2_O_2_ were added and the reaction mixture was stirred at 0 °C for 30 min. The sample was then purified with a PD-10 desalting column containing Sephadex G-25 resin to eliminate H_2_O_2_, and freeze-dried to yield oxidized-ELP (mean yield > 98%). The ^1^H NMR spectra in Figure S8 of each modified ELP were recorded in D_2_O and carefully assigned. For ELP-[*M_1_V_3_-*20*]-[I-*20*]: ^1^H NMR (400.3 MHz, CDCl_3_, 298K): δ 4.57 (m, 6H, 6 N-C*H*_Met_), 4.24 (m, 20H, 20 N-C*H*_Ile_ as guest residue), 4.18 (m, 15H, 15 N-C*H*_Val_ as guest residue), 2.99 (m, 12H, 6 C*H*_2_S), 2.74 (s, 18H, 6 SC*H*_3_), 1.49 (m, 20H, 20 N=C-NH-C*H*_2_-C*H*_3_). For ELP-[*M_1_V_3_-*40*]-[I-*20*]: ^1^H NMR (400.3 MHz, CDCl_3_, 298K): δ 4.57 (m, 11H, 11 N-C*H*_Met_), 4.24 (m, 20H, 20 N-C*H*_Ile_ as guest residue), 4.18 (m, 30H, 30 N-C*H*_Val_ as guest residue), 2.99 (m, 22H, 11 C*H*_2_S), 2.74 (s, 33H, 11 SC*H*_3_), 1.49 (m, 20H, 20 N=C-NH-C*H*_2_-C*H*_3_). For ELP-[*M_1_V_3_-*60*]-[I-*20*]: ^1^H NMR (400.3 MHz, CDCl_3_, 298K): δ 4.57 (m, 16H, 16 N-C*H*_Met_), 4.24 (m, 20H, 20 N-C*H*_Ile_ as guest residue), 4.18 (m, 45H, 45 N-C*H*_Val_ as guest residue), 2.99 (m, 32H, 16 C*H*_2_S), 2.74 (s, 48H, 16 SC*H*_3_), 1.49 (m, 20H, 20 N=C-NH-C*H*_2_-C*H*_3_). The reaction yield for the diblocks ELP-[*M_1_V_3_-*i*]-[I-90] was determined by MALDI-MS (Figure S9).

### 2.7. Characterization Techniques

#### 2.7.1. Sodium Dodecyl Sulfate-Polyacrylamide Gel Electrophoresis (SDS-PAGE) 

ELPs were separated under reducing conditions by SDS-PAGE with 4–15% Mini-PROTEIN® TGX precast protein gel (Bio-Rad) and Tris/Glycine/SDS buffer (Bio-Rad). Polypeptide bands were detected by the stain-free technique with Bio-Rad Gel Doc EZ system. Gels were also stained with Coomassie (InstantBlue^TM^, Sigma Aldrich, Saint Quentin Fallavier, France). 

#### 2.7.2. Mass Spectrometry Analysis

Mass spectrometry analysis was performed either on a MALDI-ToF-ToF (Ultraflex III, Bruker) equipped with a matrix-assisted laser desorption/ionization source or an ESI-Q-ToF (QToF Premier, Waters) equipped with an electrospray ionization source. ELP samples were prepared as follows: freeze-dried compounds were dissolved in cold water, if soluble, or solubilized in drops of DMSO and diluted in water/methanol (1/1, *v*/*v*). Final concentrations vary from 2 to 5 mg/mL. For MALDI-MS analysis, ELP samples were mixed to the matrix solution: sinapinic acid (10 mg/mL in acetonitrile/0.1% trifluoroacetic acid in water (1/1, *v*/*v*)), then, 1–2 µL of this solution were added to a metal plate. After solvent evaporation, the solid residue was exposed under laser (Smartbeam, Nd:YAG, 355 nm). Analysis was performed in positive linear mode, and a protein standards mixture was used for external calibration of the instrument in a mass range adapted to proteins of interest. For ESI-MS analysis, ELP solutions were diluted in methanol/0.1% formic acid in water (1/1 *v*/*v*), and exposed under ionization source with 5 µL/min as flow rate. Analysis was performed in positive ionization mode and external calibration was carried out with a solution of standard protein: apomyoglobin. Proteins experimental masses were obtained by deconvolution of ESI spectra with MaxEnt (Waters) software, using an algorithm based on maximal entropy approach. Alternatively, ESI-TOF analysis was performed on a Xevo G2QTOF (Waters). Freeze-dried samples were resuspended in ultrapure water to a concentration of 10 µM. All samples were acidified with 0.1% formic acid upon injection, followed by on-line fractionation on a Polaris 3 C18 column (Agilent) with water/acetonitrile gradients. Deconvoluted spectra were obtained using Mass Lynx v4.1.

#### 2.7.3. Nuclear Magnetic Resonance (NMR) 

NMR spectra of ELP-containing samples were acquired in D_2_O either at 278 K or at 289 K on a AVANCE NEO 400 BRUKER spectrometer operating at 400.3 MHz for ^1^H and equipped with a Bruker multinuclear z-gradient direct cryoprobe-head. The solvent signal was used as the reference signal. Data processing was performed using Topspin software. 

#### 2.7.4. Size-Exclusion Chromatography (SEC) 

Protein polymer dispersities were determined by size exclusion chromatography using dimethylformamide + lithium bromide (LiBr) at 1g/L as the eluent. Measurements in DMF were performed on an Ultimate 3000 system from Thermoscientific equipped with diode array detector DAD. The system also includes a multi-angles light scattering detector MALS and differential refractive index detector dRI from Wyatt technology. Polymers were separated on two KD-803 Shodex gel columns (300 × 8 mm^2^) (exclusion limits from 1000 Da to 50,000 Da) at a flow rate of 0.8 mL/min. Columns temperature was held at 50 °C. EasiVial kit of polystyrene from Agilent was used as the standard (Mn from 162 to 364,000 Da).

#### 2.7.5. Turbidity Analysis

ELP transition temperatures (Tt) were determined for different concentrations either in phosphate buffer saline (PBS) or in ultrapure water by measuring the turbidity at 350 nm between 12 and 80 °C, at 1 °C/min scan rate. Data were collected on a Cary 100 UV−Vis spectrophotometer equipped with a multi-cell thermoelectric temperature controller from Agilent Technologies. The Tt is defined as the maximum of the first derivative of absorbance versus temperature.

#### 2.7.6. Dynamic Light Scattering

Measurements were performed on a NanoZS instrument (Malvern, U.K.) at an angle of 90° (constant position). The derived count rate (DCR) was defined as the mean scattered intensity normalized by the attenuation factor. Three independent measurements of ten 10 s-runs were recorded and averaged during a heating ramp 7–37 °C.

## 3. Results and Discussion

### 3.1. Development and Thermal Study of the First Series of Diblock ELPs: ELP-[M_1_V_3_-i]-[I-20] (i = 20, 40, 60)

#### 3.1.1. Production of Diblock ELPs and of a Library of Related Monoblock ELPs

To understand the thermal behavior of ELP-[M_1_V_3_-*i*]-[I-*20*] diblocks (*i* = *20*, *40*, *60*), two libraries of individual monoblocks based on ELP-[M_1_V_3_-*i*] (*i* = *20*, *40*, *60*, *80*, *100*) and ELP-[I-*j*] (*j* = *20*, *40*, *60*) were first produced recombinantly. Genes were constructed by recursive directional ligation, inserted in *E. coli* bacteria and proteins produced from selected bacterial clones following previously reported procedures (Table 1) [41,43]. Three diblock copolymers ELP-[M_1_V_3_-*i*]-[I-*20*] (*i* = *20*, *40*, *60*) were then constructed by ligation of corresponding genes and produced in *E. coli* (Table 1). Gene sequences as well as their corresponding protein sequences are provided in supporting information (Figure S1).

After cloning of each diblock ELP-encoding gene, the proteins were expressed in *E. coli* for 21 h after induction by IPTG, extracted from cell lysates and purified by inverse transition cycling (ITC) [46], dialyzed extensively against ultrapure water and lyophilized to provide diblock ELPs with an overall yield of about 100–120 mg/L culture. The purity of each diblock ELP was assessed by SDS-PAGE which evidenced the presence of dimers resulting from intermolecular disulfide bridge formation between Cys residues at the *C*-terminal end of ELPs. (Figure S2) Mass spectrometry analysis was performed and experimental masses of 17,385 Da, 25,734 Da and 34,001 Da were found for ELP-[M_1_V_3_-*i*]-[I-*20*] (*i* = *20*, *40*, *60*), respectively, (Figure S3) in good agreement with theoretical values (i.e., 17,384 Da, 25,734 Da and 34,084 Da). The three diblock ELPs were also characterized by ^1^H NMR spectroscopy in D_2_O. (Figure S4) To avoid the presence of dimers that would complicate the interpretation of subsequent thermal studies, diblock ELPs were reduced with TCEP and the side chain of the *C*-terminal Cys residue was capped with NEM via thiol-ene Michael addition. SDS-PAGE analysis of the purified products showed that they contained only monomers of ELP-[M_1_V_3_-*i*]-[I-*20*] (*i* = *20*, *40*, *60*) (Figure S5).

#### 3.1.2. Thermal Properties of ELP-[M_1_V_3_-i]-[I-20] Diblocks and of the Monoblock Library

ELPs as thermoresponsive biopolymers are characterized by a cloud point in aqueous solution that can be determined by turbidimetry. The cloud points, also termed transition temperatures (Tts), were experimentally measured by UV-Vis spectroscopy (absorbance at 350 nm) on ELP solutions in PBS at different concentrations. The absorbance was measured at 350 nm, because this wavelength is optimal for the detection of ELP aggregation into nanoparticles and aggregates [38]. Absorbance versus temperature curves were plotted and the Tt at each concentration determined by the maximum of the first derivative. Tts versus concentration curves were then plotted and fitted with a log-linear law. Fits of the different curves allowed the determination of the different parameters of the empirical equation established by Chilkoti et al. [27]:

(1) $$T_{t}=T_{t,c}+\frac{k}{L}ln\left(\frac{C_{c}}{C}\right)$$

in which $T_{t,c}$ (°C) corresponds to the critical temperature, $C_{c}$ (µM) the critical concentration, k a constant (°C) and L the number of pentapeptide repeat units.

The resulting equations were subsequently used to simulate Tts for ELPs of similar composition but of different lengths within a concentration range from 1 µM to 1 M for monoblock ELPs and from 1 µM to 1 mM for diblock ELPs.

##### Thermal Behavior of Monoblock ELPs

**Figure 2:**
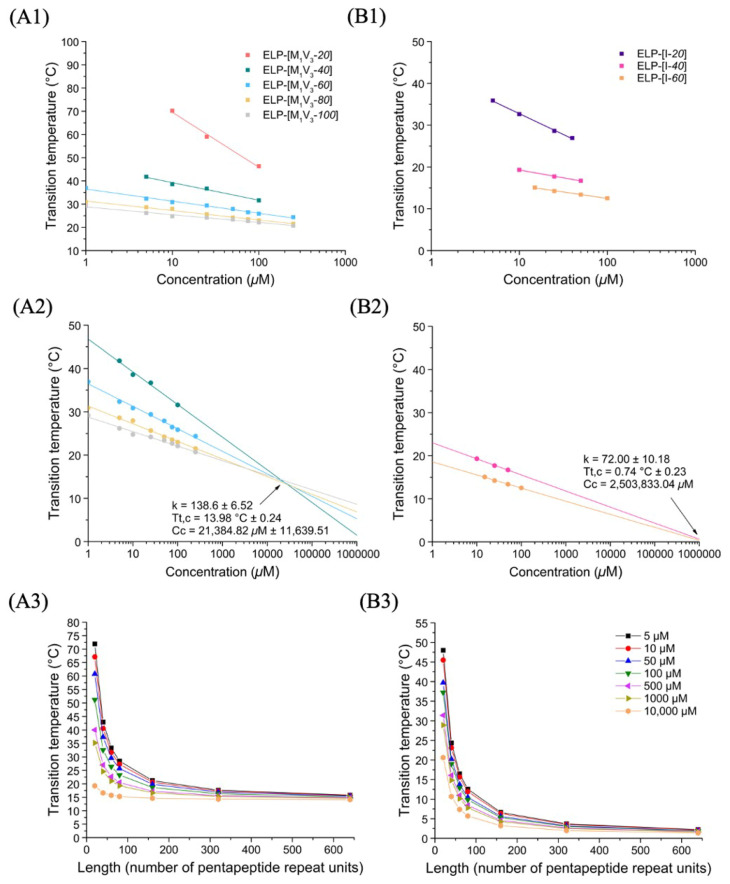
Thermal study of monoblock ELPs in PBS. (**A**) ELP-[M_1_V_3_-*i*]. (**B**) ELP-[I-*j*]. (1) Tt values as function of sample concentration and log fitted. (2) Simulation of Tt values in a larger concentration range from 1 to 1,000,000 µM allowing $T_{t,c}$ and $C_{c}$ determination. (3) *T*t values at different *I*, *j* = length (L).

Cloud points of ELP-[M_1_V_3_-*i*] (*i* = *20*, *40*, *60*, *80*, *100*) and ELP-[I-*j*] (*j* = *20*, *40*, *60*) monoblocks in PBS were measured at different concentrations, (Figure S10) and Tts plotted as a function of concentration for each series of ELP monoblocks. (Figure 2, panel A1 for ELP-[M_1_V_3_-*i*] and panel B1 for ELP-[I-*j*]). The linear dependence of Tt versus log(C) correlated well with Equation (1), and acutely fitted as R² were close to 0.99. (Table S1) For each series, Tt values were then estimated within a range from 1 µM to 1 M thanks to the fitted equations and simulated Tt values were plotted versus concentration (Figure 2, panels A2 and B2). The Tt datasets included in the linear regressions, are those of monoblocks with *i*,*j* ≥ 40 repeats. The selection of datasets was necessary to construct a model that better simulated the experimental conditions and results, similarly to previously reported models such as those described by MacKay et al. [38]. 

From this initial study, the behavior of the two series of monoblock ELPs was found to follow general trends consistent with the literature [27,38]. For both series, Tts inversely correlated with molar concentration and polypeptide length. Additionally, the longer the polypeptide, the weaker the dependance of Tt on molar concentration. The $T_{t}$ *vs.* log(C) curves depicted in Figure 2, panels A2 and B2, intersect at one unique point corresponding to the critical temperature $T_{t,c}$ and concentration $C_{c}$ defining each family of ELP with identical amino acid composition, namely ELP-[M_1_V_3_-*i*] and ELP-[I-*j*]. At this critical point ($T_{t,c}$; $C_{c}$) estimated graphically, all polypeptides with a given sequence have the same solution behavior, independently of their lengths and molar masses [38]. Consistent with the literature, ELPs containing valine and methionine as guest residues were less hydrophobic than those with isoleucine [22]. The fit equations obtained from experimental curves correlated to Equation (1) (Figure 2, panels A1-2 and B1-2) enabled to determine the mean k value for each family of ELP, which allowed us to study the dependency of $T_{t}$ on ELP length and concentration. Indeed, having determined $T_{t,c}$, $C_{c}$ and k for a family of ELP and implementing them in Equation (1), makes possible the ELP transition temperatures prediction for any concentration and length (Figure 2, panels A3 and B3). Results of the two series of monoblocks showed a similar trend in terms of length and concentration dependency. For both ELP monoblock families, depending on concentration, Tt values show an important dependence on ELP length (L in Equation (1)), especially for short lengths. However, the larger the length of the ELP, the lower the impact of concentration on Tt, which decreases significantly when L reaches about 150 repeats.

##### Thermal Behavior of ELP-[M_1_V_3_-*i*]-[I-*20*] Diblocks

**Figure 3:**
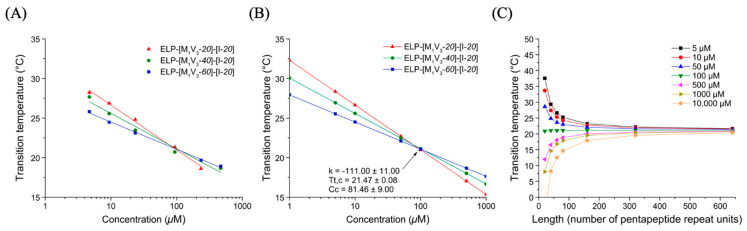
Thermal study of diblock ELP-[M_1_V_3_-*i*]-[I-*20*] (*i*= *20*, *40*, *60*) in PBS. (**A**) Tt values as function of sample concentration and log fitted. (**B**) Simulation of Tt values in a larger concentration range from 1 to 1000 µM allowing $T_{t,c}$ and $C_{c}$ determination. (**C**) *T*t values at different length (L).

In a similar manner, turbidimetry experiments were performed for the three diblock ELP-[M_1_V_3_-*i*]-[I-*20*] (*i* = *20*, *40*, *60*) in PBS at different concentrations. (Figure S11). In first assumption, it was rather surprising that none of these diblocks showed two transitions temperatures. In particular for ELP-[M_1_V_3_-*20*]-[I-*20*], whose individual blocks (i.e., ELP-[M_1_V_3_-*20*] and ELP-[I-*20*]) present very different Tts at the same concentration, one would have anticipated that both Met/Val and Ile-containing blocks would have presented individual phase transitions with two separate Tts: one consisting of a critical micelle temperature (CMT) described as the temperature at which the most hydrophobic block collapses, and the second corresponding to a bulk transition temperature T_t,bulk_ described as the temperature at which the second block collapses and leads to a coacervation process [38]. A single transition instead was observed for the three diblocks, ELP-[M_1_V_3_-*i*]-[I-*20*] (*i* = *20*, *40*, *60*), with all the hallmarks of a single block. Indeed, the Tt decreased when the concentration of the solution increased, which was consistent with general results on ELPs following Equation (1). In an attempt to compare the thermal behavior of these different diblocks, data were plotted and log-fitted (Figure 3A, Table S2), after Tts determination through the first derivative method. They were then simulated within a range of concentration from 1 to 1000 µM (Figure 3B).

The characteristic plots for diblock ELPs displayed a similar trend than monoblock ELPs: at fixed concentration and before the critical point, Tt values decreased with the increase in the block length. The critical point ($T_{t,c}$; $C_{c})$ values were determined for this family of ELP allowing the simulation of Tt for different length and concentration (Figure 3C). The temperature plots obtained through Equation (1) showed a high dependency of Tt over length, especially below 150 units. 

Despite the rationale based on Urry’s amino acid hydrophilicity classification [22] to design our diblock ELPs, these constructions did not present two separate transition temperatures and behaved as monoblock ELPs. The presence of methionine residues however allowed us to easily access and study a second family of ELPs without the design of a new ELP sequence requiring the construction of a new encoding gene and multiple molecular cloning steps. Indeed, our group has developed and applied over the past years a set of chemoselective reactions at the methionine side chain as a mean to easily tune physico-chemical properties of methionine-containing ELPs or to introduce specific pendant groups or moieties [41,42,47,48]. In particular, chemical oxidation of the thioether side chain into sulfoxide was found to have a major impact on the cloud point due to a drastic increase in hydrophilicity [49]. This strategy was therefore applied to the diblocks ELP-[M_1_V_3_-*i*]-[I-*20*] (*i* = *20*, *40*, *60*) to increase the hydrophilic character of the *N*-terminal ELP block.

### 3.2. Development and Thermal Study of the Second Series of Diblock ELPs: ELP-[*M_1_V_3_-i]-[I-20] (i = 20, 40, 60)

#### 3.2.1. Chemical Oxidation at Methionine Residues of Diblock ELPs 

**Figure 4:**
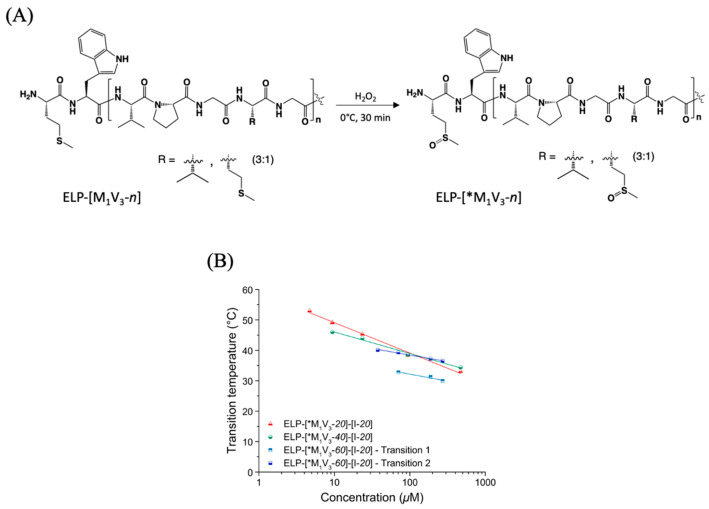
(**A**) Chemical structure of oxidized ELP. (**B**) Tt values of ELP-[*M_1_V_3_-*i*]-[I-*20*] (*i* = *20*, *40*, *60*) in ultrapure water as function of sample concentration and log fitted.

The diblock ELPs described previously were, therefore, subjected to chemical oxidation to turn all thioether groups into sulfoxide derivatives. (Figure 4A) The reaction was performed in mild conditions to avoid the formation of sulfone derivatives that are less hydrophilic.

After purification, the diblock ELPs were analyzed by ^1^H NMR spectroscopy to confirm the quantitative oxidation of thioether groups. (Figure S8) For each compound, the resonance of the methionine methyl group protons (S-C*H*_3_, noted m on spectra) at δ = 2.12 ppm in pristine ELPs (Figure S4) was shifted to δ = 2.75 ppm in sulfoxide-containing ELPs noted ELP-[*M_1_V_3_-*i*]-[I-*20*] (*i* = *20*, *40*, *60*). Similarly, the resonance of the methylene protons bonded to sulfur in methionine (S-C*H*_2_, noted l on spectra) was shifted from δ = 2.6 ppm to δ = 3 ppm. The conversion was assessed by comparing the integrals of -C*H*_3_ (m) and -C*H*_2_- (l) resonances to the unchanged resonances at δ = 4.18 ppm and 4.24 ppm corresponding to the αC*H* protons of the isoleucine and the valine located at the guest residue positions, respectively. The unchanged resonance at δ = 1.49 ppm corresponding to the methylene group of isoleucine (-C*H*_2_-) can also be used for conversion estimation. NMR analysis evidenced a complete conversion of thioether groups into sulfoxides for the three diblock ELPs, namely ELP-[*M_1_V_3_-*i*]-[I-*20*] (*i* = *20*, *40*, *60*) contained a total of 6, 11 and 16 sulfoxide groups in the *N*-terminal ELP block.

#### 3.2.2. Thermal Properties of Oxidized Diblock ELPs

Similarly, to the precedent study, transition temperatures of oxidized diblocks ELP-[*M_1_V_3_-*i*]-[I-*20*] (*i* = *20*, *40*, *60*) were measured at different concentrations in ultrapure water, (Figure S12) and were plotted as function of molar concentration (Figure 4B, Table S2). As expected from previous studies, Tts increased as compared to native diblock ELPs. In the case of ELP-[*M_1_V_3_-*i*]-[I-*20*] (*i* = *20*, *40*, *60*), the range of transition temperature moved from 20–35 °C for non-oxidized diblocks to 30–55 °C, consistent with the dependency of a diblock T_t,bulk_ with the corresponding hydrophilic block behavior. Unlike ELP-[*M_1_V_3_-*i*]-[I-*20*] (*i* = *20*, *40*), the oxidized ELP with a longer and more hydrophilic block, ELP-[*M_1_V_3_-*60*]-[I-*20*], displayed two transitions at higher concentrations starting from 75 μM meaning that a larger length and hydrophilicity of the methionine containing block are needed to generate these double transition temperatures. Thus, we need a concentration higher than 50 µM to be able to have this double transition temperature. Regarding the conclusion of MacKay’s study [38], namely that a certain length of the hydrophobic block is necessary to form stable nanoparticles, we designed a third series of diblock ELPs with a longer isoleucine-containing block with the expectation of better differentiating the transition temperatures of the two blocks.

### 3.3. Development and Thermal Study of the Third Series of Diblock ELPs: ELP-[*M_1_V_3_-i]-[I-90] (i = 40, 60)

Considering that diblock ELPs require a minimum length above which they assemble into stable particles [38], the molecular weight of the ELP-[I-*j*] block of the ELP-[M_1_V_3_-*i*]-[I-*j*] library was increased to 90 repeats. The block length was increased for the ELPs to access a thermal phase transition of three distinct regimes (unimers, nanoparticles, aggregates) after increasing the hydrophilicity of methionine [39]. Therefore, two new diblock ELPs were designed by recombinant DNA technology and produced in *E. coli*, namely ELP-[M_1_V_3_-*40*]-[I-*90*] and ELP-[M_1_V_3_-*60*]-[I-*90*] (Table 1).

#### 3.3.1. Thermal Properties of ELP-[M_1_V_3_-i]-[I-*90*] Diblock ELPs (I = 40, 60)

**Figure 5:**
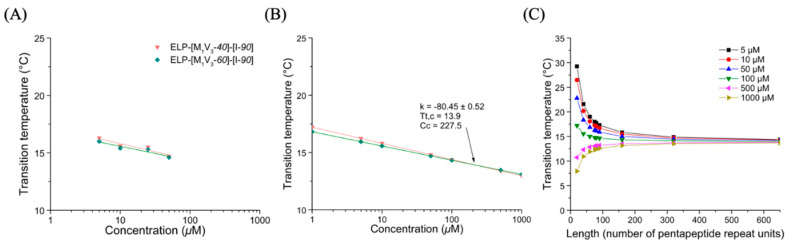
Turbidity of ELP diblocks ELP-[M_1_V_3_-*i*]-[I-*90*] in PBS. (**A**) Tt values as function of sample concentration and log fitted. (**B**) Simulation of Tt values in a larger concentration range from 1 to 1000 µM for Tt,c and Cc determination. (**C**) *T*t values at different length (L).

Before their chemical oxidation, the diblocks ELP-[M_1_V_3_-*i*]-[I-*90*] (*i* = 40, 60) showed the same self-assembling property as their shorter counterparts, with an unimer-to-aggregate transition. These two diblocks exhibited a single Tt in the range of 13–17 °C as determined by turbidity experiments (Figure 5A,B, Figure S13, Table S2). The decrease in the Tt values when compared to those of the shorter ELP-[*M_1_V_3_-*i*]-[I-*20*] (*i* = *20*, *40*, *60*) was a consequence of the significant molecular weight increase. Furthermore, the introduction of more hydrophobic VPGIG repeats in the design of this library led to a more restricted variation of the Tt as a function of the diblock length (Figure 5C) when compared to the ELP-[M_1_V_3_-*i*]-[I-*20*].

#### 3.3.2. Chemoselective Modification of ELP-[*M_1_V_3_-i]-[I-*90*] Methionine Residues 

The methionine hydrophilicity was increased via chemoselective oxidation of its thioether into sulfoxide. The reaction was conducted using hydrogen peroxide as mentioned earlier. MALDI-TOF experiments confirmed the conversion of most thioethers into sulfoxides. Two extra oxygen atoms were detected from the molar mass difference between the native ELPs and their oxidized versions, suggesting that two thioether groups were converted into sulfones for both ELP-[*M_1_V_3_-*40*]-[I-*90*] and ELP-[*M_1_V_3_-*60*]-[I-*90*] (Table 1, Figure S9). In particular, the molecular weight of ELP-[*M_1_V_3_-*40*]-[I-*90*] was 55,522 Da instead of the expected 55,491 Da, which corresponds to an addition of 13 oxygen atoms instead of 11. In the case of ELP-[*M_1_V_3_-*60*]-[I-*90*], molecular weight of the diblock was 63,949 Da instead of 63,921 Da, i.e., 18 oxygen atoms instead of 16. 

#### 3.3.3. Thermal Properties of Oxidized Diblocks ELP-[*M_1_V_3_-i]-[I-90] 

**Figure 6:**
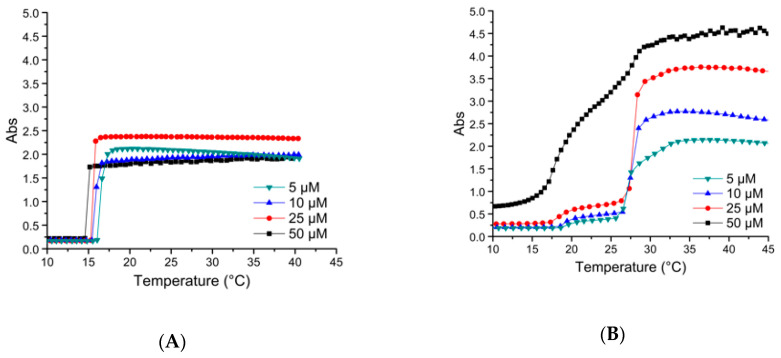
Turbidity assays at 350 nm with a rate of 1 °C/min of (**A**) ELP-[M_1_V_3_-*40*]-[I-*90*] and (**B**) its oxidized counterpart, ELP-[*M_1_V_3_-*40*]-[I-*90*], in PBS. The results demonstrate the appearance of a second transition temperature which was induced by modifying the methionine thioether.

**Figure 7:**
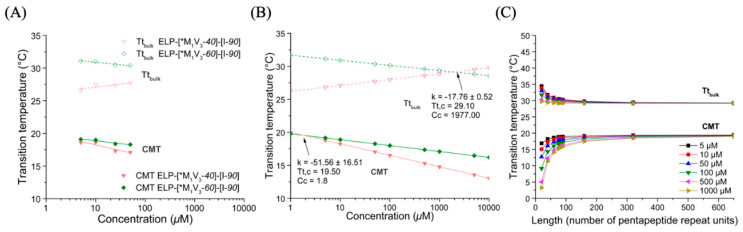
Turbidity of ELP diblocks ELP-[*M_1_V_3_-*i*]-[I-*90*] in PBS. (**A**) CMT and T_t,bulk_ values as function of sample concentration and log fitted. (**B**) Simulation of CMT and T_t,bulk_ values in a larger concentration range from 1 to 1000 µM for Tt,c and Cc determination. (**C**) CMT and T_t,bulk_ values at different length (L).

The conversion of the thioether into sulfoxide induced a change in the thermal behavior of the diblocks, as was found by turbidity measurements (Figure 6 and Figure S13). The non-modified diblocks exhibited only one sharp increase in turbidity at their transition temperature, similar to the shorter diblocks (ELP-[M_1_V_3_-*i*]-[I-*20*]). After the sulfoxide modification, the turbidity increased in two steps, each corresponding to the collapse of the hydrophobic and hydrophilic block at the CMT and T_t,bulk_, respectively. ELP-[*M_1_V_3_-*40*]-[I-*90*] and ELP-[*M_1_V_3_-*60*]-[I-*90*] exhibited a first collapse of the hydrophobic block at a CMT range between 17–20 °C, and a second collapse of the hydrophilic block at a T_t,bulk_ range of 26–31 °C (Figure 7, Figure S13, Table S2).

Notably, the T_t,bulk_ values of the ELP-[*M_1_V_3_-*60*]-[I-*90*] were larger than those of the ELP-[*M_1_V_3_-*40*]-[I-*90*], which indicates that the increased hydrophilic-to-hydrophobic ratio favors the stability of the nanoparticle phase in a wider temperature range. The T_t,bulk_-concentration curve of the ELP-[*M_1_V_3_-*40*]-[I-*90*] had a positive slope, which may be attributed to an artifact due to the existence of the sulfone-oxidized residues.

**Figure 8:**
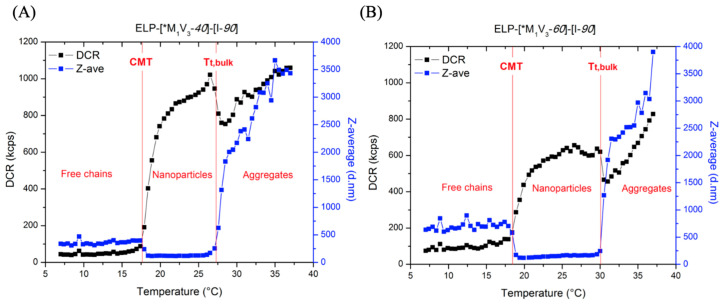
DLS measurement of 10 µM (**A**) ELP-[*M_1_V_3_-*40*]-[I-*90*] and (**B**) ELP-[*M_1_V_3_-*60*]-[I-*90*] in PBS on a thermal ramp 7–40 °C (DCR: Derived Count Rate, the parameter representative of the scattering intensity. Z-ave: the Z-average value, i.e., the intensity-weighted mean hydrodynamic size of the particles ensemble).

The thermal behavior of the ELP-[M_1_V_3_-*i*]-[I-*90*] diblocks was further evaluated by DLS (Figure 8). At temperatures below the CMT the scattering intensity is low, which may be attributed to protein unimers coexisting with a small number of aggregates. Between the CMT and T_t,bulk_, the scattering intensity increased due to the formation of nanoparticles possessing a diameter of 120–160 nm and desolvation of their cores [39]. A similar size range was previously found for particles formed by ELP diblocks comprised of a glutamic acid and an alanine block [50,51]. The aggregates present below the CMT scatter more light compared to the ELP nanoparticles, therefore the average size decreased in the nanoparticles phase. The particles formed by ELP-[M_1_V_3_-*60*]-[I-*90*] were less polydisperse in size compared to the shorter diblock (Figure S14A,B), which indicates that the aggregation number and stability depend on the hydrophilic-to-hydrophobic ratio. Above the T_t,bulk_, the regime is characterized by the formation of micron-sized aggregates. The transition temperatures detected by DLS were the same as those detected by turbidimetry (CMT, T_t,bulk_ = 18.5 °C, 27 °C for ELP-[*M_1_V_3_-*40*]-[I-*90*] and 19 °C, 31 °C for ELP-[*M_1_V_3_-*60*]-[I-*90*] at 10 µM, respectively).

The self-assembly propensity of the ELP-[M_1_V_3_-*i*]-[I-*90*] diblocks is similar to that of ELP diblocks developed for therapeutic applications [52]. The formation of nanoparticles contributes to the increased circulation time and enhanced internalization by cells [53], while the aggregates are useful as therapeutic depots [54]. Regarding the transition temperatures, different chemoselective modifications of the methionine residue can be applied to achieve nanoparticle formation at body temperature [49].

## 4. Conclusions

With the goal of forming nanoparticles from ELP diblock copolymers based on ELP-[M_1_V_3_-*i*]-[I-*j*], an investigation of ELP-[M_1_V_3_-*i*]-[I-*20*] (*i* = *20*, *40*, *60*), was first performed. More precisely, their specific thermal behavior was characterized. Despite a rational design based on Urry’s amino acid hydrophilicity classification [22], the study showed that the diblock systems did not present two but a single transition temperature, behaving as ELP monoblocks. The presence of methionine residues within our diblock ELPs enabled us to increase the hydrophilicity of the ELP-[M_1_V_3_-*i*] block via a simple chemical oxidation of the thioether side chain into sulfoxide. However, results displayed two transitions temperature only for the oxidized ELP with a longer and more hydrophilic block ELP-[*M_1_V_3_-*60*]-[I-*20*], requiring a minimum concentration of 75 µM. Thus, ELPs with larger lengths were designed, namely ELP-[M_1_V_3_-*40*]-[I-*90*] and ELP-[M_1_V_3_-*60*]-[I-*90*] to access a thermal phase transition with three distinct regimes (unimers, micelles, aggregates). Prior to any chemical modification, the two diblocks had a similar self-assembling propensity as their shorter counterparts (unimer-to-aggregate transition). However, after chemical oxidation of methionine residues, the thermal behavior of these diblocks exhibited a first collapse of the hydrophobic block at a CMT (17–20 °C), followed by a second collapse of the hydrophilic block at a T_t,bulk_ (26–31 °C). By tuning chemically ELP-[M_1_V_3_-*i*]-[I-*j*] diblocks, via oxidation here, we have been able to generate a first transition temperature, CMT, correlated to stable micelle formation as demonstrated by dynamic light scattering analysis. Thus, chemoselective modifications serve as a precise, easy and fast tool to tune the thermal behavior of ELP diblocks leading to specific structures formation. 

## Figures and Tables

**Table 1:** Characteristics of monoblock and diblock libraries.

ELP Notation	Amino Acid Sequence	Theoretical MW (Da)	Experimental ^a^ MW (Da)
*Monoblock library*			
ELP-[M_1_V_3_-*20*]	MW[VPGVGVPGMG(VPGVG)_2_]_5_	8685	8686
ELP-[M_1_V_3_-*40*]	MW[VPGVGVPGMG(VPGVG)_2_]_10_	17,035	17,035
ELP-[M_1_V_3_-*60*]	MW[VPGVGVPGMG(VPGVG)_2_]_15_	25,385	25,387
ELP-[M_1_V_3_-*80*]	MW[VPGVGVPGMG(VPGVG)_2_]_20_	33,735	33,737
ELP-[M_1_V_3_-*100*]	MW[VPGVGVPGMG(VPGVG)_2_]_25_	42,085	42,088
ELP-[I-*20*]	MW[VPGIG]_20_C(*N*-EtSucc) ^b^	9034	9034
ELP-[I-*40*]	MW[VPGIG]_40_C(*N*-EtSucc) ^b^	17,504	17,504
ELP-[I-*60*]	MW[VPGIG]_60_C(*N*-EtSucc) ^b^	25,974	25,974
*Diblock library*			
ELP-[M_1_V_3_-*20*]-[I-*20*]	MW[VPGVGVPGMG(VPGVG)_2_]_5_[VPGIG]_20_C(*N*-EtSucc) ^b^	17,384	n.d. ^c^
ELP-[M_1_V_3_-*40*]-[I-*20*]	MW[VPGVGVPGMG(VPGVG)_2_]_10_[VPGIG]_20_C(*N*-EtSucc) ^b^	25,734	n.d. ^c^
ELP-[M_1_V_3_-*60*]-[I-*20*]	MW[VPGVGVPGMG(VPGVG)_2_]_15_[VPGIG]_20_C(*N*-EtSucc) ^b^	34,084	n.d. ^c^
ELP-[*M_1_V_3_-*20*]-[I-*20*] ^d^	M(O)W[VPGVGVPGM(O)G(VPGVG)_2_]_5_[IGVPG]_20_C(*N*-EtSucc) ^b,d^	17,480	n.d. ^e^
ELP-[*M_1_V_3_-*40*]-[I-*20*] ^d^	M(O)W[VPGVGVPGM(O)G(VPGVG)_2_]_10_[VPGIG]_20_C(*N*-EtSucc) ^b,d^	25,910	n.d. ^e^
ELP-[*M_1_V_3_-*60*]-[I-*20*] ^d^	M(O)W[VPGVGVPGM(O)G(VPGVG)_2_]_15_[VPGIG]_20_C(*N*-EtSucc) ^b,d^	34,340	n.d. ^e^
ELP-[M_1_V_3_-*40*]-[I-*90*]	MW[VPGVGVPGMG(VPGVG)_2_]_10_[IGVPG]_90_Y	55,315	55,314
ELP-[M_1_V_3_-*60*]-[I-*90*]	MW[VPGVGVPGMG(VPGVG)_2_]_15_[IGVPG]_90_Y	63,665	63,664
ELP-[*M_1_V_3_-*40*]-[I-*90*] ^d^	M(O)W[VPGVGVPGM(O)G(VPGVG)_2_]_10_[IGVPG]_90_Y	55,491	55,523 ^f^
ELP-[*M_1_V_3_-*60*]-[I-*90*] ^d^	M(O)W[VPGVGVPGM(O)G(VPGVG)_2_]_15_[IGVPG]_90_Y	63,921	63,949 ^f^

^a^ Determined by Mass Spectrometry (ESI or MALDI); ^b^ Thiol side chain of *C*-terminal Cys residue capped with *N*-ethylmaleimide. ^c^ Molar mass of these diblock ELPs were assessed before reaction with *N-*Ethylmaleimide; ^d^ Thioether side chain of Met residues oxidized into sulfoxide with H_2_O_2_. *M designates oxidized M (i.e., M(O)); ^e^ As calculated from ^1^H NMR analyses; ^f^ Two Met residues were found oxidized into sulfone (M(O_2_)).

## Data Availability

The data presented in this study are available on request from the corresponding author.

## Supplementary Materials

The following are available online at https://www.mdpi.com/article/10.3390/polym13091470/s1.
