# Supplementary material for: Refining the Design of Diblock Elastin-Like Polypeptides for Self-Assembly into Nanoparticles
Source: Polymers (Basel). 2021 May 1;13(9):1470. doi: 10.3390/polym13091470 (PMC8125372; doi:10.3390/polym13091470)
Supplement: Supplementary file 1 [file polymers-13-01470-s001.zip › polymers-1170130-supplementary.pdf]

Supporting Information for

Refining the design of diblock elastin-like polypeptides for self-assembly into nanoparticles

Michèle Dai,[§] Evangelos Georgilis,[§] Guillaume Goudounet, Bertrand Garbay, Jan Pille, Jan C. M. van Hest, Xavier Schultze, Elisabeth Garanger* and Sébastien Lecommandoux*

[§] Authors contributed equally to the work.

Gene and protein sequences of ELP-[M_1V_3-*i*]-[I-*20*] (*i = 20, 40, 60*) diblocks

ELP-[M_1V_3-*20*]-[I-*20*]

```
atgtgggttccaggcgttggagtgccaggcatgggcgtaccaggtgtgggagttccaggt
 M  W  V  P  G  V  G  V  P  G  M  G  V  P  G  V  G  V  P  G
gttggggtaccgggcgtcggagttcctgggatgggagttccgggagttggtgtgccgggt
 V  G  V  P  G  V  G  V  P  G  M  G  V  P  G  V  G  V  P  G
gtcggtgtgcctggggtgggtgttccaggtatgggggttccgggtgtcggcgttcccggc
 V  G  V  P  G  V  G  V  P  G  M  G  V  P  G  V  G  V  P  G
gttggtgttccaggcgtaggtgtgccgggaatgggggttccgggagttggtgtacctggc
 V  G  V  P  G  V  G  V  P  G  M  G  V  P  G  V  G  V  P  G
gtgggagtacctggagtcggcgtgcctggtatgggcgtgcctggcgtcggcgtacctggc
 V  G  V  P  G  V  G  V  P  G  M  G  V  P  G  V  G  V  P  G
gtaggtgttccaggcattggagtgccaggcattggcgtaccaggtattggagttccaggt
 V  G  V  P  G  I  G  V  P  G  I  G  V  P  G  I  G  V  P  G
attggggtaccgggcatcggagttcctgggatcggagttccgggaattggtgtgccgggt
 I  G  V  P  G  I  G  V  P  G  I  G  V  P  G  I  G  V  P  G
atcggtgtgcctgggatcggtgttccaggtatcggggttccgggtatcggcgttcccggc
 I  G  V  P  G  I  G  V  P  G  I  G  V  P  G  I  G  V  P  G
attggtgttccaggcatcggtgtgccgggaattggggttccggggattggtgtacctggc
 I  G  V  P  G  I  G  V  P  G  I  G  V  P  G  I  G  V  P  G
attggggtacctggaatcggcgtgcctggtattggcgtgcctggcatcggcgttcctggc
 I  G  V  P  G  I  G  V  P  G  I  G  V  P  G  I  G  V  P  G
attggttgctaa
 I  G  C  -
```

ELP-[M_1V_3-*40*]-[I-*20*]

```
atgtgggttccaggcgttggagtgccaggcatgggcgtaccaggtgtgggagttccaggt
 M  W  V  P  G  V  G  V  P  G  M  G  V  P  G  V  G  V  P  G
Gttggggtaccgggcgtcggagttcctgggatgggagttccgggagttggtgtgccgggt
 V  G  V  P  G  V  G  V  P  G  M  G  V  P  G  V  G  V  P  G
gtcggtgtgcctggggtgggtgttccaggtatgggggttccgggtgtcggcgttcccggc
 V  G  V  P  G  V  G  V  P  G  M  G  V  P  G  V  G  V  P  G
gttggtgttccaggcgtaggtgtgccgggaatgggggttccgggagttggtgtacctggc
 V  G  V  P  G  V  G  V  P  G  M  G  V  P  G  V  G  V  P  G
gtgggagtacctggagtcggcgtgcctggtatgggcgtgcctggcgtcggcgtacctggc
 V  G  V  P  G  V  G  V  P  G  M  G  V  P  G  V  G  V  P  G
gtaggtgttccaggcgttggagtgccaggcatgggcgtaccaggtgtgggagttccaggt
 V  G  V  P  G  V  G  V  P  G  M  G  V  P  G  V  G  V  P  G
gttggggtaccgggcgtcggagttcctgggatgggagttccgggagttggtgtgccgggt
 V  G  V  P  G  V  G  V  P  G  M  G  V  P  G  V  G  V  P  G
gtcggtgtgcctggggtgggtgttccaggtatgggggttccgggtgtcggcgttcccggc
 V  G  V  P  G  V  G  V  P  G  M  G  V  P  G  V  G  V  P  G
gttggtgttccaggcgtaggtgtgccgggaatgggggttccgggagttggtgtacctggc
 V  G  V  P  G  V  G  V  P  G  M  G  V  P  G  V  G  V  P  G
gtgggagtacctggagtcggcgtgcctggtatgggcgtgcctggcgtcggcgtacctggc
 V  G  V  P  G  V  G  V  P  G  M  G  V  P  G  V  G  V  P  G
gtaggtgttccaggcattggagtgccaggcattggcgtaccaggtattggagttccaggt
 V  G  V  P  G  I  G  V  P  G  I  G  V  P  G  I  G  V  P  G
attggggtaccgggcatcggagttcctgggatcggagttccgggaattggtgtgccgggt
```

```
 I  G  V  P  G  I  G  V  P  G  I  G  V  P  G  I  G  V  P  G
atcggtgtgcctgggatcggtgttccaggtatcggggttccgggtatcggcgttcccggc
 I  G  V  P  G  I  G  V  P  G  I  G  V  P  G  I  G  V  P  G
attggtgttccaggcatcggtgtgccgggaattggggttccggggattggtgtacctggc
 I  G  V  P  G  I  G  V  P  G  I  G  V  P  G  I  G  V  P  G
attggggtacctggaatcggcgtgcctggtattggcgtgcctggcatcggcgttcctggc
 I  G  V  P  G  I  G  V  P  G  I  G  V  P  G  I  G  V  P  G
attggttgctaa
 I  G  C  -
```

ELP-[M_1V_3-*60*]-[I-*20*]

```
atgtgggttccaggcgttggagtgccaggcatgggcgtaccaggtgtgggagttccaggt
 M  W  V  P  G  V  G  V  P  G  M  G  V  P  G  V  G  V  P  G
gttggggtaccgggcgtcggagttcctgggatgggagttccgggagttggtgtgccgggt
 V  G  V  P  G  V  G  V  P  G  M  G  V  P  G  V  G  V  P  G
gtcggtgtgcctggggtgggtgttccaggtatgggggttccgggtgtcggcgttcccggc
 V  G  V  P  G  V  G  V  P  G  M  G  V  P  G  V  G  V  P  G
gttggtgttccaggcgtaggtgtaccgggaatgggggttccgggagttggtgtacctggc
 V  G  V  P  G  V  G  V  P  G  M  G  V  P  G  V  G  V  P  G
gtgggagtacctggagtcggcgtgcctggtatgggcgtgcctggcgtcggcgtacctggc
 V  G  V  P  G  V  G  V  P  G  M  G  V  P  G  V  G  V  P  G
gtaggtgttccaggcgttggagtgccaggcatgggcgtaccaggtgtgggagttccaggt
 V  G  V  P  G  V  G  V  P  G  M  G  V  P  G  V  G  V  P  G
gttggggtaccgggcgtcggagttcctgggatgggagttccgggagttggtgtgccgggt
 V  G  V  P  G  V  G  V  P  G  M  G  V  P  G  V  G  V  P  G
gtcggtgtgcctggggtgggtgttccaggtatgggggttccgggtgtcggcgttcccggc
 V  G  V  P  G  V  G  V  P  G  M  G  V  P  G  V  G  V  P  G
gttggtgttccaggcgtaggtgtgccgggaatgggggttccgggagttggtgtacctggc
 V  G  V  P  G  V  G  V  P  G  M  G  V  P  G  V  G  V  P  G
gtgggagtacctggagtcggcgtgcctggtatgggcgtgcctggcgtcggcgtacctggc
 V  G  V  P  G  V  G  V  P  G  M  G  V  P  G  V  G  V  P  G
gtaggtgttccaggcgttggagtgccaggcatgggcgtaccaggtgtgggagttccaggt
 V  G  V  P  G  V  G  V  P  G  M  G  V  P  G  V  G  V  P  G
gttggggtaccgggcgtcggagttcctgggatgggagttccgggagttggtgtgccgggt
 V  G  V  P  G  V  G  V  P  G  M  G  V  P  G  V  G  V  P  G
gtcggtgtgcctggggtgggtgttccaggtatgggggttccgggtgtcggcgttcccggc
 V  G  V  P  G  V  G  V  P  G  M  G  V  P  G  V  G  V  P  G
gttggtgttccaggcgtaggtgtgccgggaatgggggttccgggagttggtgtacctggc
 V  G  V  P  G  V  G  V  P  G  M  G  V  P  G  V  G  V  P  G
gtgggagtacctggagtcggcgtgcctggtatgggcgtgcctggcgtcggcgtacctggc
 V  G  V  P  G  V  G  V  P  G  M  G  V  P  G  V  G  V  P  G
gtaggtgttccaggcattggagtgccaggcattggcgtaccaggtattggagttccaggt
 V  G  V  P  G  I  G  V  P  G  I  G  V  P  G  I  G  V  P  G
attggggtaccgggcatcggagttcctgggatcggagttccgggaattggtgtgccgggt
 I  G  V  P  G  I  G  V  P  G  I  G  V  P  G  I  G  V  P  G
atcggtgtgcctgggatcggtgttccaggtatcggggttccgggtatcggcgttcccggc
 I  G  V  P  G  I  G  V  P  G  I  G  V  P  G  I  G  V  P  G
attggtgttccaggcatcggtgtgccgggaattggggttccggggattggtgtacctggc
 I  G  V  P  G  I  G  V  P  G  I  G  V  P  G  I  G  V  P  G
attggggtacctggaatcggcgtgcctggtattggcgtgcctggcatcggcgttcctggc
```

```
 I   G   V   P   G   I   G   V   P   G   I   G   V   P   G   I   G   V   P   G
attggttgctaa
 I   G   C   -
```

Figure S1. Nucleotide sequences of the ELP-[M_1V_3-*i*]-[I-*20*] (*i* = *20*, *40*, *60*) genes and corresponding amino acids sequences.

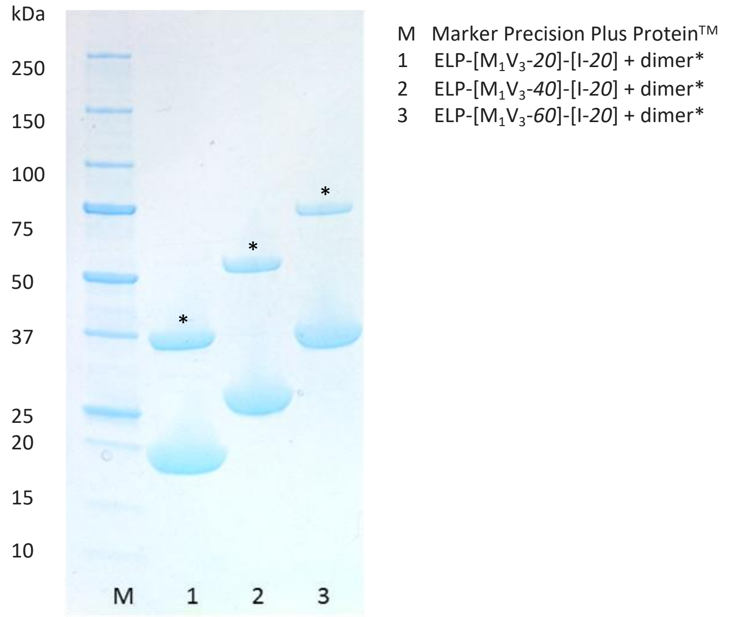

Figure S2. Coomassie-stained SDS-PAGE gel of purified ELP-[M_1V_3-*i*]-[I-*20*] (*i* = *20*, *40*, *60*) before capping the cysteines located at the C-terminal of the different ELP diblocks (with their respective dimers ELP-[M_1V_3-*i*]-[I-*20*]-[I-*20*]-[M_1V_3-*i*]).

(A)

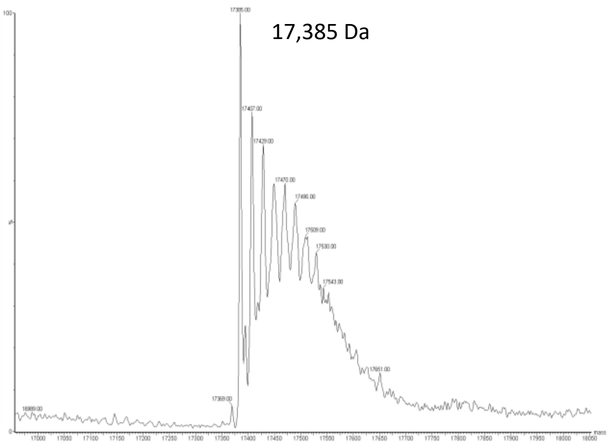

(B)

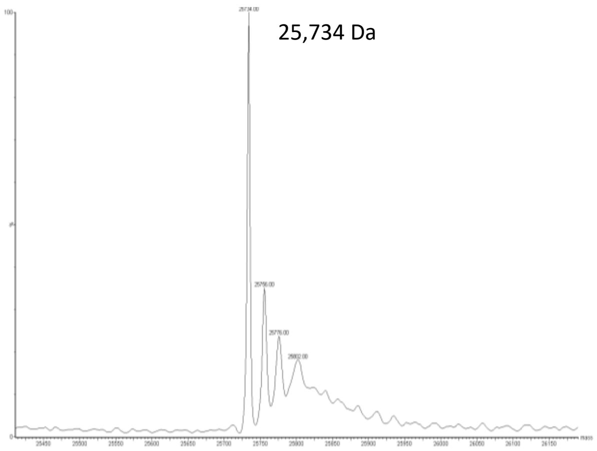

(C)

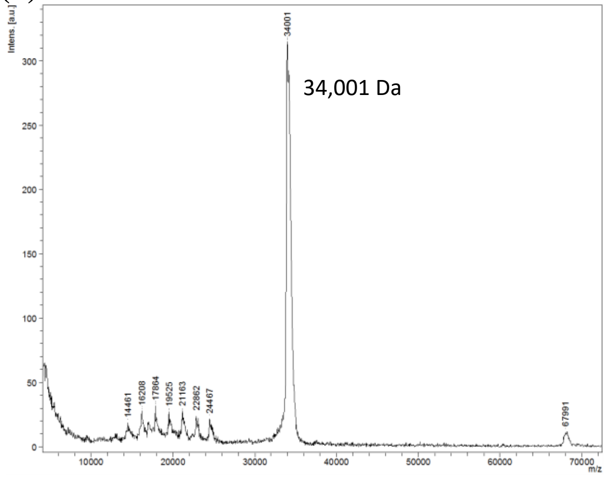

Figure S3. Mass *spectra* of (A) *ESI-MS of non-capped ELP-[M_1V_3-*20*]-[I-*20*], (B) ESI of non-capped ELP-[M_1V_3-*40*]-[I-*20*], (C) **MALDI-TOF *spectrum* of non-capped ELP-[M_1V_3-*60*]-[I-*20*]. *ESI-MS = electrospray ionization mass *spectrum*. **MALDI-TOF = Matrix Assisted *Laser* Desorption Ionization-time-of-flight.

(A)

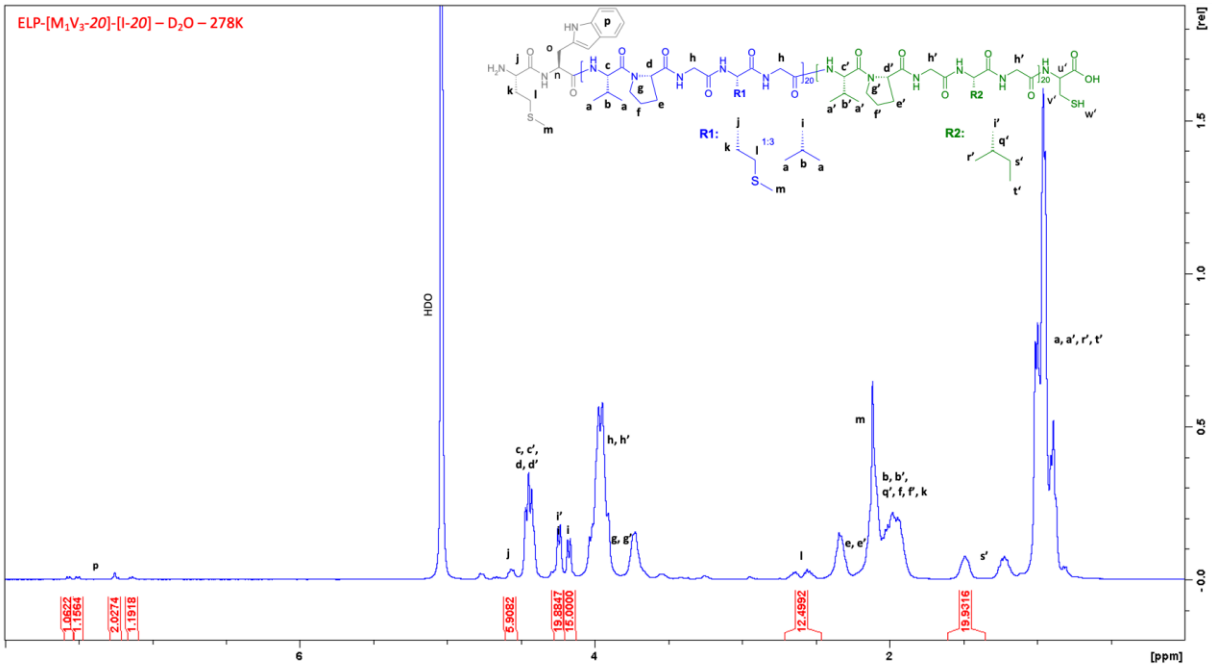

(B)

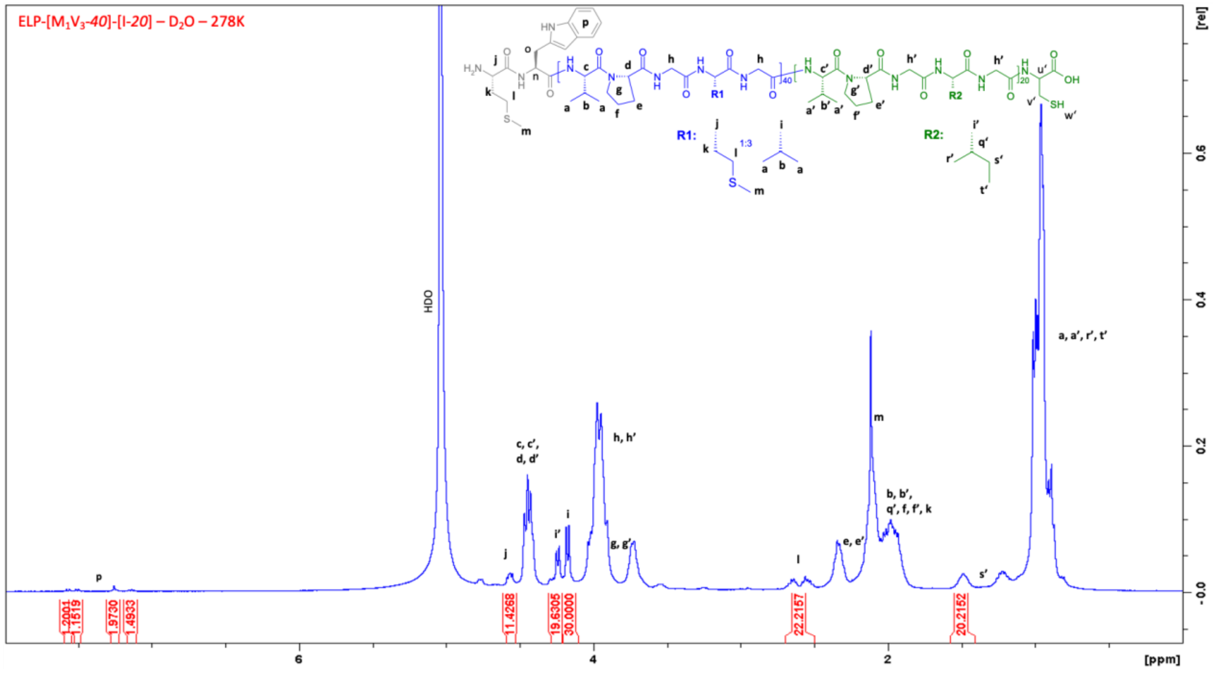

(C)

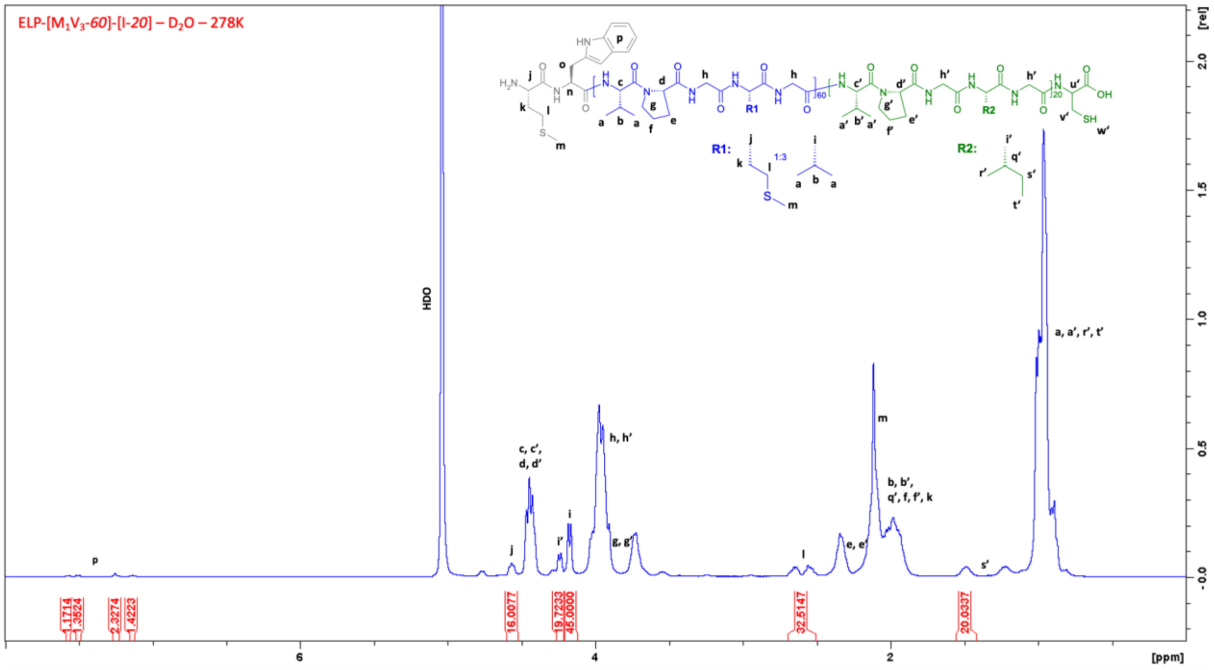

Figure S4. NMR *spectra* in D_2O of non-capped (A) ELP-[M_1V_3-*20*]-[I-*20*], (B) ELP-[M_1V_3-*40*]-[I-*20*] and (C) ELP-[M_1V_3-*60*]-[I-*20*].

Cysteines from the diblocks ELP-[M_1V_3-*i*]-[I-*20*] (*i* = *20*, *40*, *60*) were modified to avoid disulfide bridge formation. The reducing agent tris(2-carboxyethyl)phosphine hydrochloride (TCEP·HCl) was added to break every S-S bond between cysteines and *N*-ethyl maleimide (NEM) was introduced by thiol-Michael addition in basic conditions with *N*-ethyldiisopropylamine (DIPEA) (Figure S5A). After isolation by extensive dialysis against ultrapure water and freeze-drying, the products were characterized as ELP-[M_1V_3-*i*]-[I-*20*] (*i* = *20*, *40*, *60*) by SDS-PAGE (Figure S5B)

and size-exclusion chromatography (SEC) in DMF (Figure S5C) showing both disappearance of dimer traces. To be consistent, the monoblocks ELP-[I-*j*] (*j* = *20*, *40*, *60*) were also synthetized with the same procedure than for the diblocks. As ELP-[M_1V_3-*i*] do not have cysteine residues, no modification was performed.

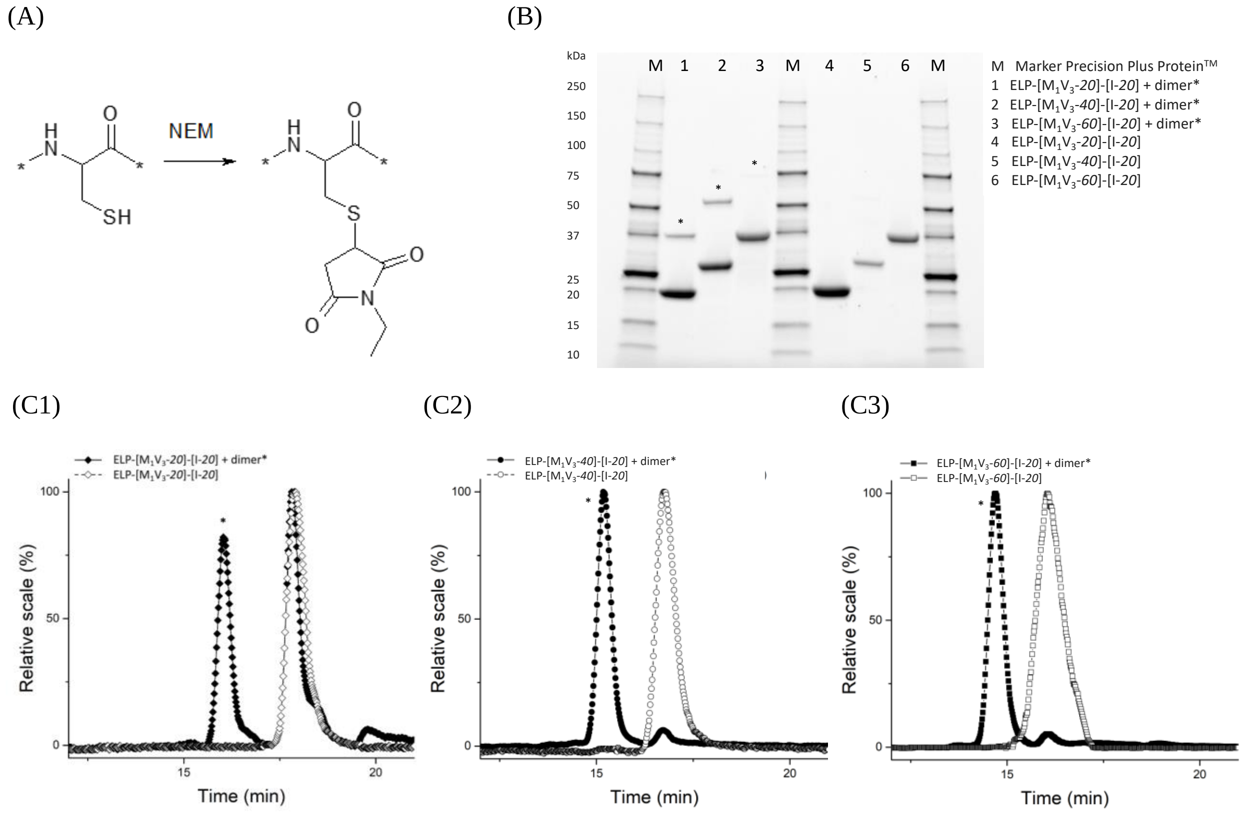

Figure S5. (A) Scheme of the introduction of a *N*-ethyl maleimide (NEM) into cysteine thiol *via* thiol-Michael addition. (B) Stain-free gel SDS-PAGE of produced diblock elastin-like polypeptides (1-2-3), and after introducing *N*-ethyl maleimide (4-5-6). (C) ELP-[M_1V_3-*i*]-[I-*20*] (*i* = *20* (C1), *40* (C2), *60* (C3)) size-exclusion chromatography *spectra* in DMF.

(A) Gene and protein sequences of ELP-[M_1V_3-*i*]-[I-*90*] (*i* = *40*, *60*) diblocks

ELP-[M_1V_3-*40*]-[I-*90*]

```
atgtgggttccaggcgttggagtgccaggcatgggcgtaccaggtgtgggagttccaggt
 M  W  V  P  G  V  G  V  P  G  M  G  V  P  G  V  G  V  P  G
gttgggtaccgggcgtcggagttcctgggatgggagttccgggagttggtgtgccgggt
 V  G  V  P  G  V  G  V  P  G  M  G  V  P  G  V  G  V  P  G
gtcggtgtgcctggggtgggtgttccaggtatgggggttccgggtgtcggcgttcccggc
 V  G  V  P  G  V  G  V  P  G  M  G  V  P  G  V  G  V  P  G
gttggtgttccaggcgtaggtgtgccgggaatgggggttccgggagttggtgtacctggc
 V  G  V  P  G  V  G  V  P  G  M  G  V  P  G  V  G  V  P  G
gtgggagtacctggagtcggcgtgcctggtatgggcgtgcctggcgtcggcgtacctggc
 V  G  V  P  G  V  G  V  P  G  M  G  V  P  G  V  G  V  P  G
gtaggtgttccaggcgttggagtgccaggcatgggcgtaccaggtgtgggagttccaggt
 V  G  V  P  G  V  G  V  P  G  M  G  V  P  G  V  G  V  P  G
gttgggtaccgggcgtcggagttcctgggatgggagttccgggagttggtgtgccgggt
 V  G  V  P  G  V  G  V  P  G  M  G  V  P  G  V  G  V  P  G
```

```
gtcggtgtgcctggggtgggtgttccaggtatgggggttccgggtgtcggcgttcccggc
 V  G  V  P  G  V  G  V  P  G  M  G  V  P  G  V  G  V  P  G
gttggtgttccaggcgtaggtgtgccgggaatgggggttccgggagttggtgtacctggc
 V  G  V  P  G  V  G  V  P  G  M  G  V  P  G  V  G  V  P  G
gtgggagtacctggagtcggcgtgcctggtatgggcgtgcctggcgtcggcgtacctggc
 V  G  V  P  G  V  G  V  P  G  M  G  V  P  G  V  G  V  P  G
gtaggcatcggtgtaccgggtatcggtgtgccgggcatcggtgtgccgggtatcggggtt
 V  G  I  G  V  P  G  I  G  V  P  G  I  G  V  P  G  I  G  V
ccaggcatcggtgtaccaggcatcggggtaccgggtatcggtgtgccgggcatcggtgtg
 P  G  I  G  V  P  G  I  G  V  P  G  I  G  V  P  G  I  G  V
ccgggtatcggggttccaggcatcggtgtaccaggcatcggtgtaccgggtatcggtgtg
 P  G  I  G  V  P  G  I  G  V  P  G  I  G  V  P  G  I  G  V
ccgggcatcggtgtgccgggtatcggggttccaggcatcggtgtaccaggcatcggtgta
 P  G  I  G  V  P  G  I  G  V  P  G  I  G  V  P  G  I  G  V
ccgggtatcggtgtgccgggcatcggtgtgccgggtatcggggttccaggcatcggtgta
 P  G  I  G  V  P  G  I  G  V  P  G  I  G  V  P  G  I  G  V
ccaggcatcggggtaccgggtatcggtgtgccgggcatcggtgtgccgggtatcggggtt
 P  G  I  G  V  P  G  I  G  V  P  G  I  G  V  P  G  I  G  V
ccaggcatcggtgtaccaggcatcggtgtaccgggtatcggtgtgccgggcatcggtgtg
 P  G  I  G  V  P  G  I  G  V  P  G  I  G  V  P  G  I  G  V
ccgggtatcggggttccaggcatcggtgtaccaggcatcggtgtaccgggtatcggtgtg
 P  G  I  G  V  P  G  I  G  V  P  G  I  G  V  P  G  I  G  V
ccgggcatcggtgtgccgggtatcggggttccaggcatcggtgtaccaggcatcggggta
 P  G  I  G  V  P  G  I  G  V  P  G  I  G  V  P  G  I  G  V
ccgggtatcggtgtgccgggcatcggtgtgccgggtatcggggttccaggcatcggtgta
 P  G  I  G  V  P  G  I  G  V  P  G  I  G  V  P  G  I  G  V
ccaggcatcggtgtaccgggtatcggtgtgccgggcatcggtgtgccgggtatcggggtt
 P  G  I  G  V  P  G  I  G  V  P  G  I  G  V  P  G  I  G  V
ccaggcatcggtgtaccaggcatcggtgtaccgggtatcggtgtgccgggcatcggtgtg
 P  G  I  G  V  P  G  I  G  V  P  G  I  G  V  P  G  I  G  V
ccgggtatcggggttccaggcatcggtgtaccaggcatcggggtaccgggtatcggtgtg
 P  G  I  G  V  P  G  I  G  V  P  G  I  G  V  P  G  I  G  V
ccgggcatcggtgtgccgggtatcggggttccaggcatcggtgtaccaggcatcggtgta
 P  G  I  G  V  P  G  I  G  V  P  G  I  G  V  P  G  I  G  V
ccgggtatcggtgtgccgggcatcggtgtgccgggtatcggggttccaggcatcggtgta
 P  G  I  G  V  P  G  I  G  V  P  G  I  G  V  P  G  I  G  V
ccaggcatcggtgtaccgggtatcggtgtgccgggcatcggtgtgccgggtatcggggtt
 P  G  I  G  V  P  G  I  G  V  P  G  I  G  V  P  G  I  G  V
ccaggcatcggtgtaccaggcatcggggtaccgggtatcggtgtgccgggcatcggtgtg
 P  G  I  G  V  P  G  I  G  V  P  G  I  G  V  P  G  I  G  V
ccgggtatcggggttccaggcatcggtgtaccaggcatcggtgtaccgggtatcggtgtg
 P  G  I  G  V  P  G  I  G  V  P  G  I  G  V  P  G  I  G  V
ccgggcatcggtgtgccgggtatcggggttccaggcatcggtgtaccaggcatcggtgta
 P  G  I  G  V  P  G  I  G  V  P  G  I  G  V  P  G  I  G  V
ccgggtatcggtgtgccgggcatcggtgtgccgggtatcggggttccaggcatcggtgta
 P  G  I  G  V  P  G  I  G  V  P  G  I  G  V  P  G  I  G  V
ccaggcatcggggtaccgggtatcggtgtgccgggcatcggtgtgccgggtatcggggtt
 P  G  I  G  V  P  G  I  G  V  P  G  I  G  V  P  G  I  G  V
ccaggcatcggtgtaccaggcatcggtgtaccgggtatcggtgtgccgggcatcggtgtg
 P  G  I  G  V  P  G  I  G  V  P  G  I  G  V  P  G  I  G  V
ccgggtatcggggttccaggcatcggtgtaccaggctactgataatga
```

```
 P  G  I  G  V  P  G  I  G  V  P  G  Y  -  -  -
```

ELP-[M_1V_3-*60*]-[I-*90*]

```
atgtgggttccaggcgttggagtgccaggcatgggcgtaccaggtgtgggagttccaggt
 M  W  V  P  G  V  G  V  P  G  M  G  V  P  G  V  G  V  P  G
gttggggtaccgggcgtcggagttcctgggatgggagttccgggagttggtgtgccgggt
 V  G  V  P  G  V  G  V  P  G  M  G  V  P  G  V  G  V  P  G
gtcggtgtgcctggggtgggtgttccaggtatgggggttccgggtgtcggcgttcccggc
 V  G  V  P  G  V  G  V  P  G  M  G  V  P  G  V  G  V  P  G
gttggtgttccaggcgtaggtgtgccgggaatgggggttccgggagttggtgtacctggc
 V  G  V  P  G  V  G  V  P  G  M  G  V  P  G  V  G  V  P  G
gtgggagtacctggagtcggcgtgcctggtatgggcgtgcctggcgtcggcgtacctggc
 V  G  V  P  G  V  G  V  P  G  M  G  V  P  G  V  G  V  P  G
gtaggtgttccaggcgttggagtgccaggcatgggcgtaccaggtgtgggagttccaggt
 V  G  V  P  G  V  G  V  P  G  M  G  V  P  G  V  G  V  P  G
gttggggtaccgggcgtcggagttcctgggatgggagttccgggagttggtgtgccgggt
 V  G  V  P  G  V  G  V  P  G  M  G  V  P  G  V  G  V  P  G
gtcggtgtgcctggggtgggtgttccaggtatgggggttccgggtgtcggcgttcccggc
 V  G  V  P  G  V  G  V  P  G  M  G  V  P  G  V  G  V  P  G
gttggtgttccaggcgtaggtgtgccgggaatgggggttccgggagttggtgtacctggc
 V  G  V  P  G  V  G  V  P  G  M  G  V  P  G  V  G  V  P  G
gtgggagtacctggagtcggcgtgcctggtatgggcgtgcctggcgtcggcgtacctggc
 V  G  V  P  G  V  G  V  P  G  M  G  V  P  G  V  G  V  P  G
gtaggcgtacctggagtcggcgtgcctggtatgggcgtgcctggcgtcggcgtacctggc
 V  G  V  P  G  V  G  V  P  G  M  G  V  P  G  V  G  V  P  G
gtaggcgtacctggagtcggcgtgcctggtatgggcgtgcctggcgtcggcgtacctggc
 V  G  V  P  G  V  G  V  P  G  M  G  V  P  G  V  G  V  P  G
gtaggcgtacctggagtcggcgtgcctggtatgggcgtgcctggcgtcggcgtacctggc
 V  G  V  P  G  V  G  V  P  G  M  G  V  P  G  V  G  V  P  G
gtaggcgtacctggagtcggcgtgcctggtatgggcgtgcctggcgtcggcgtacctggc
 V  G  V  P  G  V  G  V  P  G  M  G  V  P  G  V  G  V  P  G
gtaggcgtacctggagtcggcgtgcctggtatgggcgtgcctggcgtcggcgtacctggc
 V  G  V  P  G  V  G  V  P  G  M  G  V  P  G  V  G  V  P  G
gtaggcatcggtgtaccgggtatcggtgtgccgggcatcggtgtgccgggtatcggggtt
 V  G  I  G  V  P  G  I  G  V  P  G  I  G  V  P  G  I  G  V
ccaggcatcggtgtaccaggcatcggggtaccgggtatcggtgtgccgggcatcggtgtg
 P  G  I  G  V  P  G  I  G  V  P  G  I  G  V  P  G  I  G  V
ccgggtatcggggttccaggcatcggtgtaccaggcatcggtgtaccgggtatcggtgtg
 P  G  I  G  V  P  G  I  G  V  P  G  I  G  V  P  G  I  G  V
ccgggcatcggtgtgccgggtatcggggttccaggcatcggtgtaccaggcatcggtgta
 P  G  I  G  V  P  G  I  G  V  P  G  I  G  V  P  G  I  G  V
ccgggtatcggtgtgccgggcatcggtgtgccgggtatcggggttccaggcatcggtgta
 P  G  I  G  V  P  G  I  G  V  P  G  I  G  V  P  G  I  G  V
ccaggcatcggggtaccgggtatcggtgtgccgggcatcggtgtgccgggtatcggggtt
 P  G  I  G  V  P  G  I  G  V  P  G  I  G  V  P  G  I  G  V
ccaggcatcggtgtaccaggcatcggtgtaccgggtatcggtgtgccgggcatcggtgtg
 P  G  I  G  V  P  G  I  G  V  P  G  I  G  V  P  G  I  G  V
ccgggtatcggggttccaggcatcggtgtaccaggcatcggtgtaccgggtatcggtgtg
 P  G  I  G  V  P  G  I  G  V  P  G  I  G  V  P  G  I  G  V
ccgggcatcggtgtgccgggtatcggggttccaggcatcggtgtaccaggcatcggggta
```

```
 P  G  I  G  V  P  G  I  G  V  P  G  I  G  V  P  G  I  G  V
ccgggtatcggtgtgccgggcatcggtgtgccgggtatcggggttccaggcatcggtgta
 P  G  I  G  V  P  G  I  G  V  P  G  I  G  V  P  G  I  G  V
ccaggcatcggtgtaccgggtatcggtgtgccgggcatcggtgtgccgggtatcggggtt
 P  G  I  G  V  P  G  I  G  V  P  G  I  G  V  P  G  I  G  V
ccaggcatcggtgtaccaggcatcggtgtaccgggtatcggtgtgccgggcatcggtgtg
 P  G  I  G  V  P  G  I  G  V  P  G  I  G  V  P  G  I  G  V
ccgggtatcggggttccaggcatcggtgtaccaggcatcggggtaccgggtatcggtgtg
 P  G  I  G  V  P  G  I  G  V  P  G  I  G  V  P  G  I  G  V
ccgggcatcggtgtgccgggtatcggggttccaggcatcggtgtaccaggcatcggtgta
 P  G  I  G  V  P  G  I  G  V  P  G  I  G  V  P  G  I  G  V
ccgggtatcggtgtgccgggcatcggtgtgccgggtatcggggttccaggcatcggtgta
 P  G  I  G  V  P  G  I  G  V  P  G  I  G  V  P  G  I  G  V
ccaggcatcggtgtaccgggtatcggtgtgccgggcatcggtgtgccgggtatcggggtt
 P  G  I  G  V  P  G  I  G  V  P  G  I  G  V  P  G  I  G  V
ccaggcatcggtgtaccaggcatcggggtaccgggtatcggtgtgccgggcatcggtgtg
 P  G  I  G  V  P  G  I  G  V  P  G  I  G  V  P  G  I  G  V
ccgggtatcggggttccaggcatcggtgtaccaggcatcggtgtaccgggtatcggtgtg
 P  G  I  G  V  P  G  I  G  V  P  G  I  G  V  P  G  I  G  V
ccgggcatcggtgtgccgggtatcggggttccaggcatcggtgtaccaggcatcggtgta
 P  G  I  G  V  P  G  I  G  V  P  G  I  G  V  P  G  I  G  V
ccgggtatcggtgtgccgggcatcggtgtgccgggtatcggggttccaggcatcggtgta
 P  G  I  G  V  P  G  I  G  V  P  G  I  G  V  P  G  I  G  V
ccaggcatcggggtaccgggtatcggtgtgccgggcatcggtgtgccgggtatcggggtt
 P  G  I  G  V  P  G  I  G  V  P  G  I  G  V  P  G  I  G  V
ccaggcatcggtgtaccaggcatcggtgtaccgggtatcggtgtgccgggcatcggtgtg
 P  G  I  G  V  P  G  I  G  V  P  G  I  G  V  P  G  I  G  V
ccgggtatcggggttccaggcatcggtgtaccaggctactgataatga
 P  G  I  G  V  P  G  I  G  V  P  G  Y  -  -  -
```

(B)

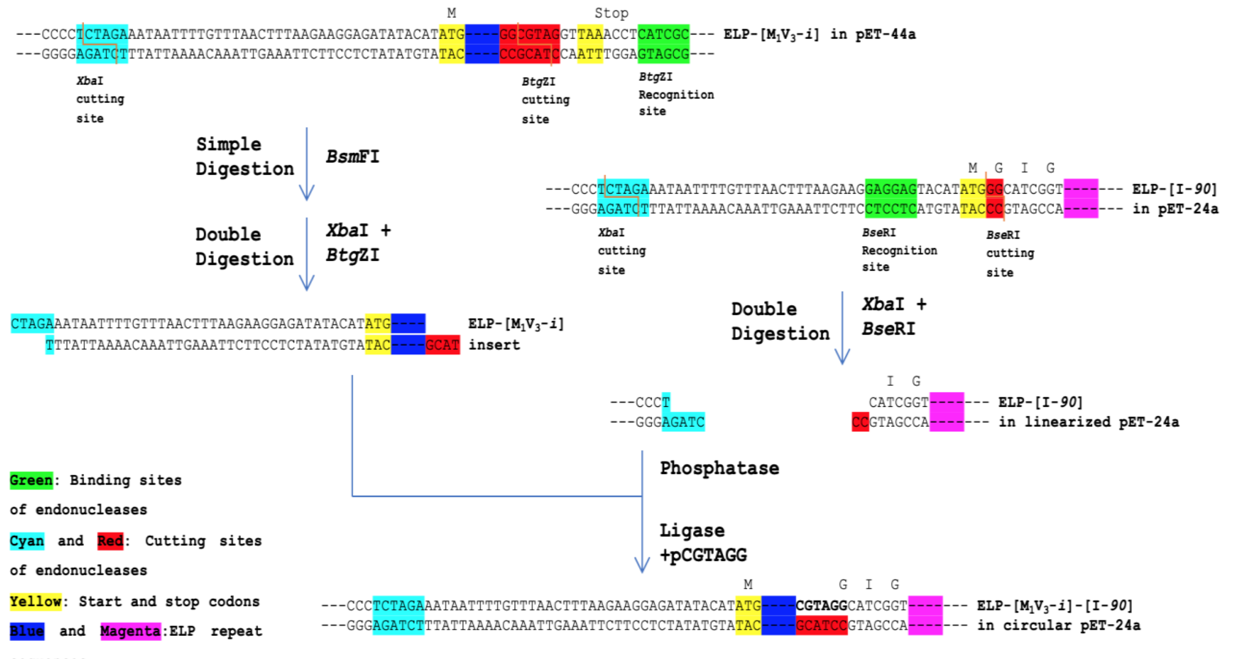

Figure S6. (A) Sequences of the ELP-[M_1V_3-*i*]-[I-*20*] (*i* = *20*, *40*, *60*) genes and of their corresponding proteins. (B) Schematic representation of the cloning strategy followed for the construction of the ELP-[M_1V_3-*i*]-[I-*90*] library.

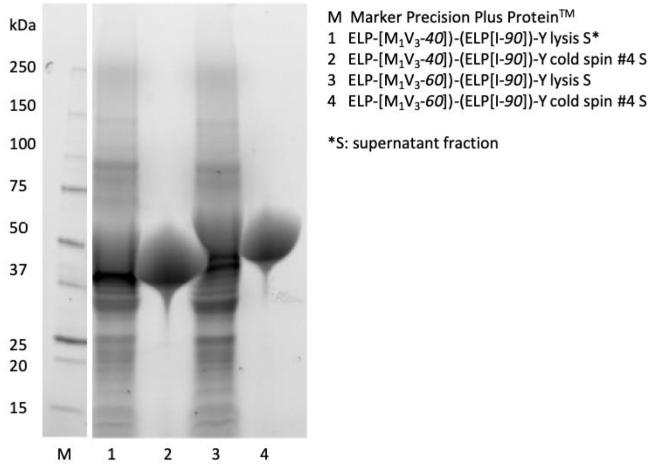

Figure S7. Coomassie-stained SDS-PAGE gel of produced ELP-[M_1V_3-*i*]-[I-*90*] (*i* = *40*, *60*).

(A)

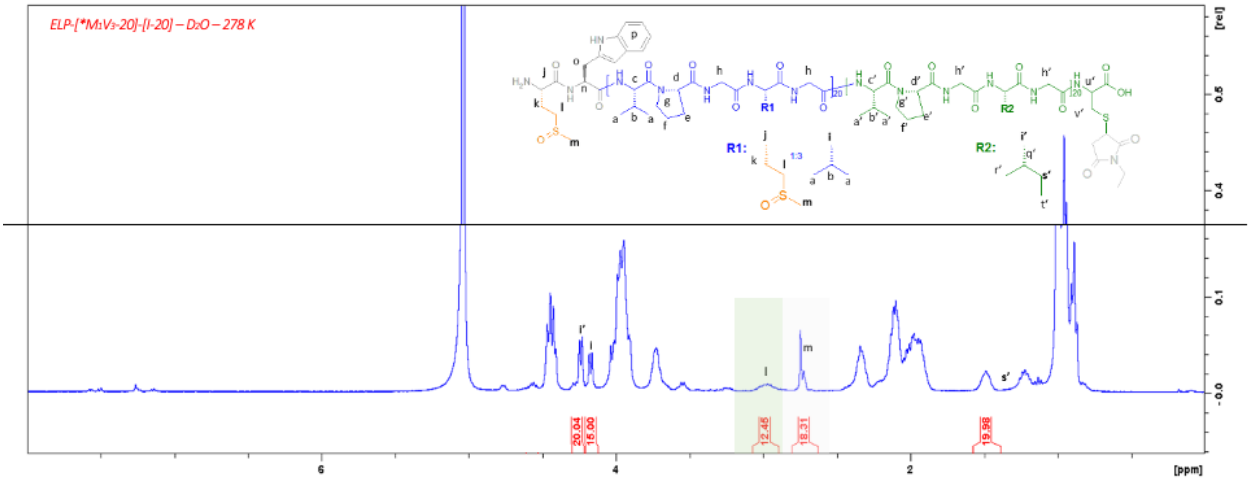

(B)

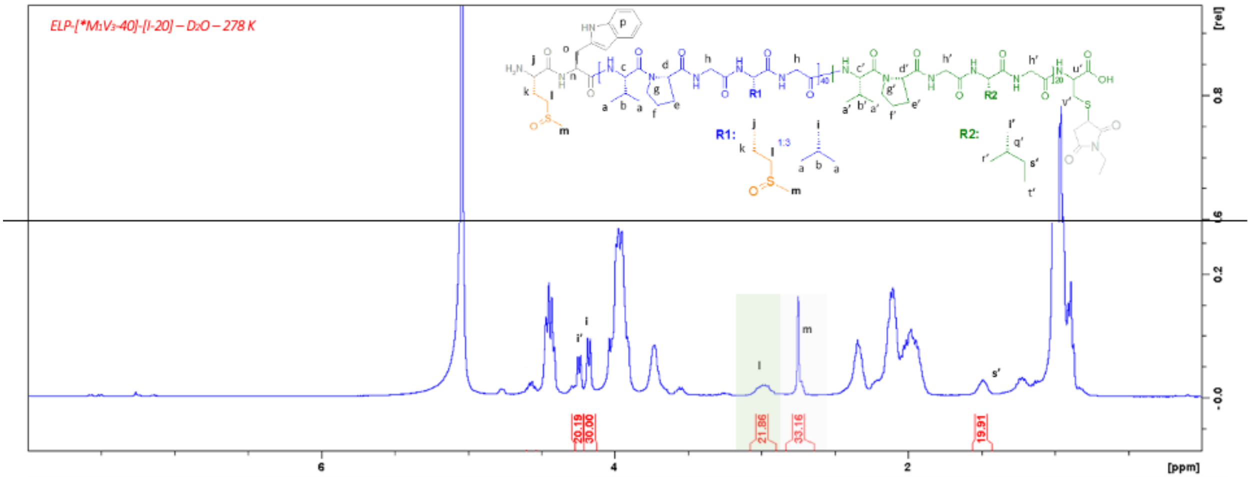

(C)

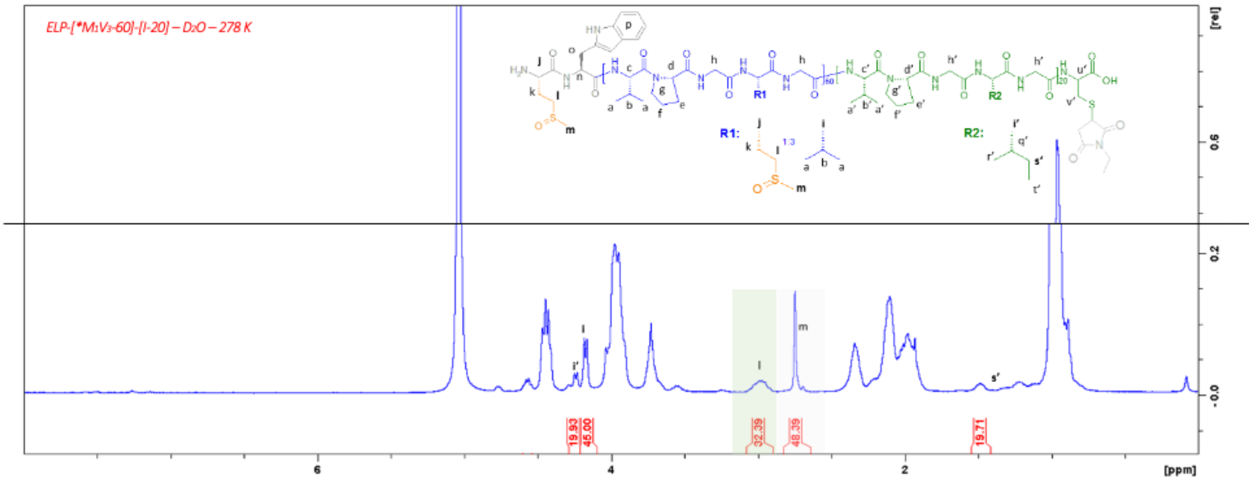

Figure S8. NMR *spectra* in D_2O of **(A)** ELP-[*M_1V_3-*20*]- [I-*20*] **(B)** ELP-[*M_1V_3-*40*]-[I-*20*] **(C)** ELP-[M_1V_3-*60*]- [I-*20*]. *A part of the spectra was removed for a better view on interesting peaks*. Green and gray boxes highlight resonances for CH_2 and CH_3 bonded to sulfur of the methionine, respectively.

(A)

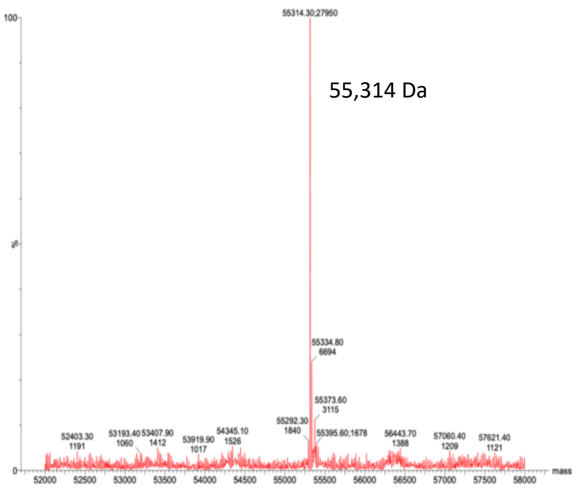

(B)

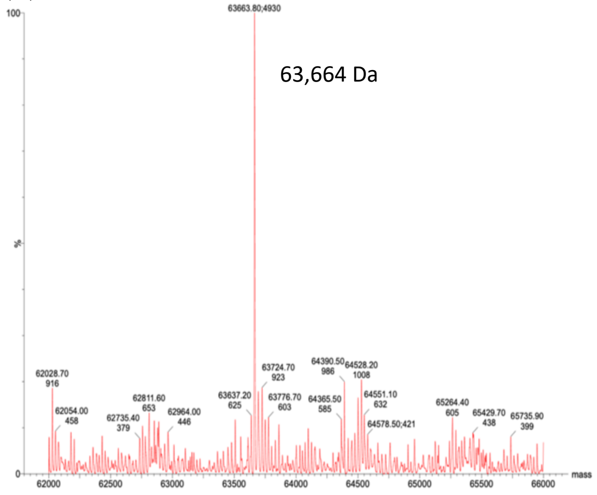

(C)

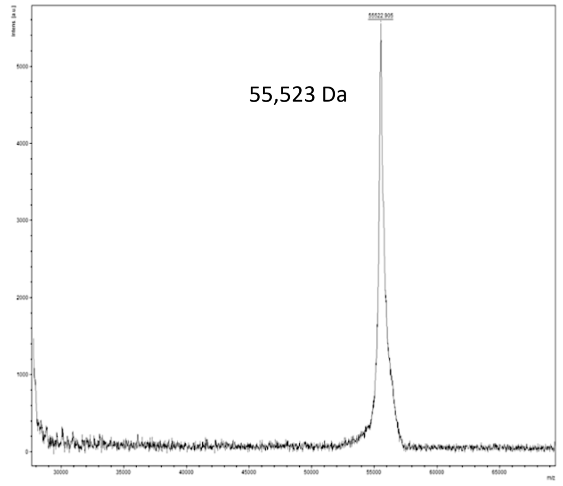

(D)

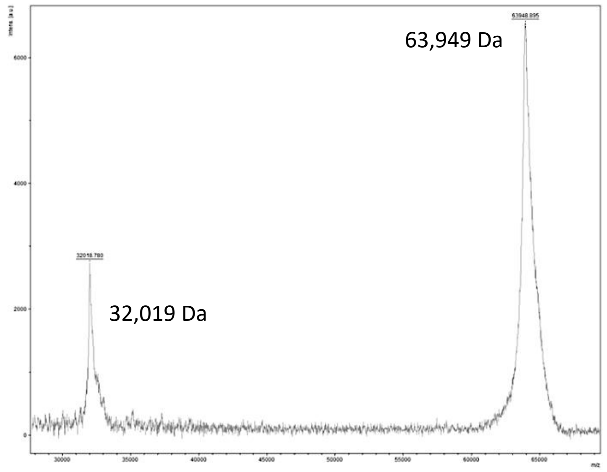

Figure S9. Mass *spectra* of (A) *ESI-MS of ELP-[M_1V_3-*40*]-[I-*90*], (B) *ESI-MS of ELP-[M_1V_3-*60*]-[I-*90*], (C)

**MALDI-TOF of ELP-[*M_1V_3-*40*]-[I-*90*] and (D) **MALDI-TOF of ELP-[*M_1V_3-*60*]-[I-*90*]. *ESI-MS = electrospray ionization mass *spectrum*. **MALDI-TOF = Matrix Assisted *Laser* Desorption Ionization-time-of-flight.

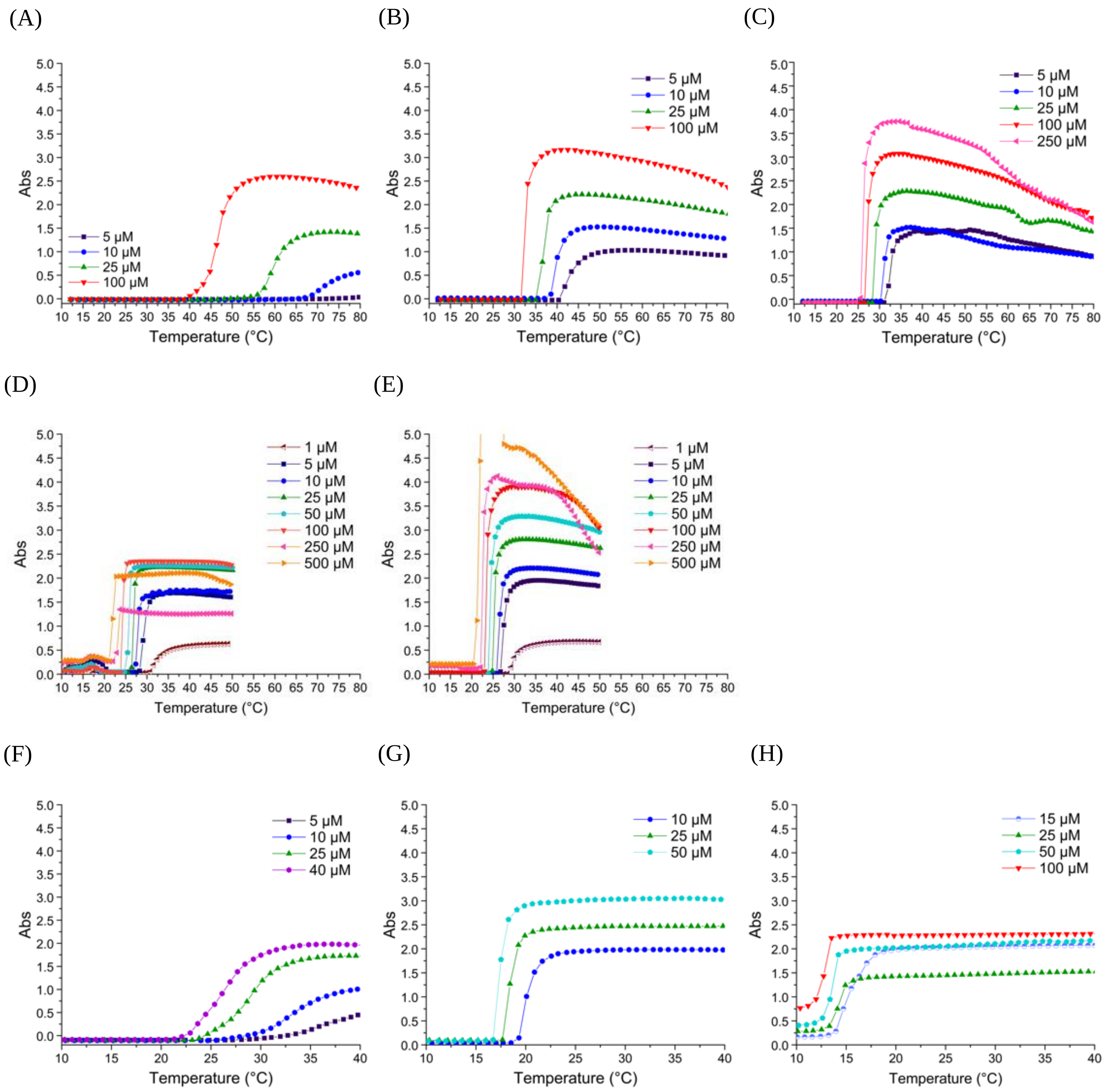

Figure S10. Turbidity assays at 350 nm with a rate of 1°C/min of (A, B, C, D, E) ELP-[M_1V_3-*i*] (*i = 20*, *40*, *60*, *80*, *100* respectively) and (F, G, H) ELP-[I-*j*] (*j = 20*, *40*, *60* respectively) in PBS.

Table S1. The linear dependence of Tt *versus* log(C) correlated to eq. 1, for ELP monoblocks experimental data.

Monoblock name	Tt *versus* log(C)	R^2
ELP-[M_1V_3-*20*]	y = -10.28*ln(x) + 93.20	0.99
ELP-[M_1V_3-*40*]	y = -3.28*ln(x) + 46.79	0.99
ELP-[M_1V_3-*60*]	y = -2.26*ln(x) + 36.48	0.99
ELP-[M_1V_3-*80*]	y = -1.77*ln(x) + 31.32	0.99
ELP-[M_1V_3-*100*]	y = -1.46*ln(x) + 28.79	0.99
ELP-[I-*20*]	y = -4.33*ln(x) + 42.71	0.99
ELP-[I-*40*]	y = -1.62*ln(x) + 23.00	0.99
ELP-[I-*60*]	y = -1.32*ln(x) + 18.58	0.99

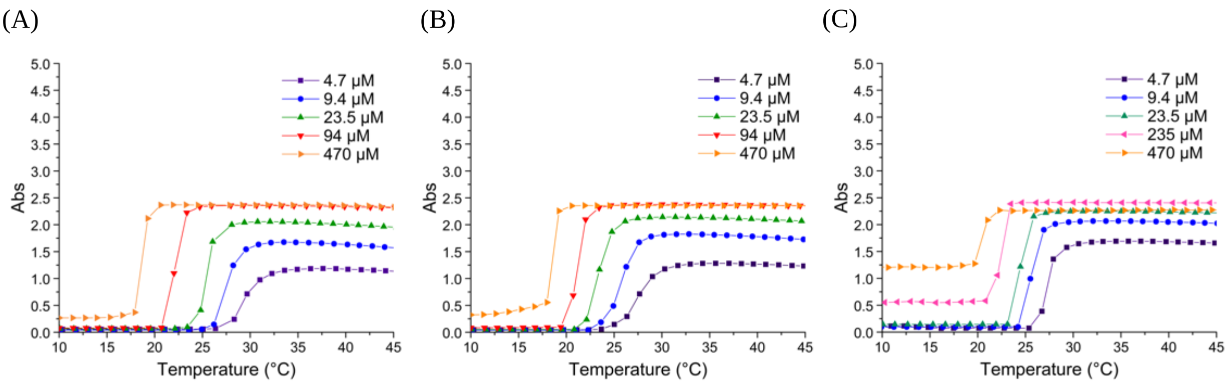

For temperatures below Tt when the chain length of the hydrophilic block was long and the solution was concentrated, absorbance was increased. This was possibly caused by the existence of some aggregates favored by the increased concentration. Similarly, the turbidity of solution above their Tt, were higher when the concentration of the solution was raised. The same reason can explain this phenomenon: the higher the concentration of the solution, the more objects were formed and the more turbid the solution will be.

Figure S11. Turbidity assays at 350 nm with a rate of 1°C/min of (A, B, C) ELP-[M$_1$V$_3$-*i*]- [I-*20*] (*i* = *20*, *40*, *60* respectively) in PBS.

Table S2. The linear dependence of Tt *versus* log(C) correlated to eq. 1, for ELP diblocks experimental data.

Monoblock name	Tt *versus* log(C)	R^2
ELP-[M$_1$V$_3$-*20*]-[I-*20*]	y = -2.45*ln(x) + 32.29	0.99
ELP-[M$_1$V$_3$-*40*]-[I-*20*]	y = -1.94*ln(x) + 30.05	0.98
ELP-[M$_1$V$_3$-*60*]-[I-*20*]	y = -1.49*ln(x) + 27.93	0.99
ELP-[*M$_1$V$_3$-*20*]-[I-*20*]	y = -4.34*ln(x) + 59.37	0.99
ELP-[*M$_1$V$_3$-*40*]-[I-*20*]	y = -3.07*ln(x) + 53.29	0.99
ELP-[*M$_1$V$_3$-*60*]-[I-*20*]	y = -1.87*ln(x) + 47.06 (Tt_{bulk})	0.99
ELP-[*M$_1$V$_3$-*60*]-[I-*20*]	y = -1.99*ln(x) + 41.35 (CMT)	0.95
ELP-[M$_1$V$_3$-*40*]-[I-*90*]	y = -0.61*ln(x) + 17.22	0.90
ELP-[M$_1$V$_3$-*60*]-[I-*90*]	y = -0.54*ln(x) + 16.82	0.91
ELP-[*M$_1$V$_3$-*40*]-[I-*90*]	y = -0.38*ln(x) + 26.22 (Tt_{bulk})	0.65
ELP-[*M$_1$V$_3$-*40*]-[I-*90*]	y = -0.76*ln(x) + 19.99 (CMT)	0.89
ELP-[*M$_1$V$_3$-*60*]-[I-*90*]	y = -0.34*ln(x) + 31.68 (Tt_{bulk})	0.94
ELP-[*M$_1$V$_3$-*60*]-[I-*90*]	y = -0.39*ln(x) + 19.78 (CMT)	0.93

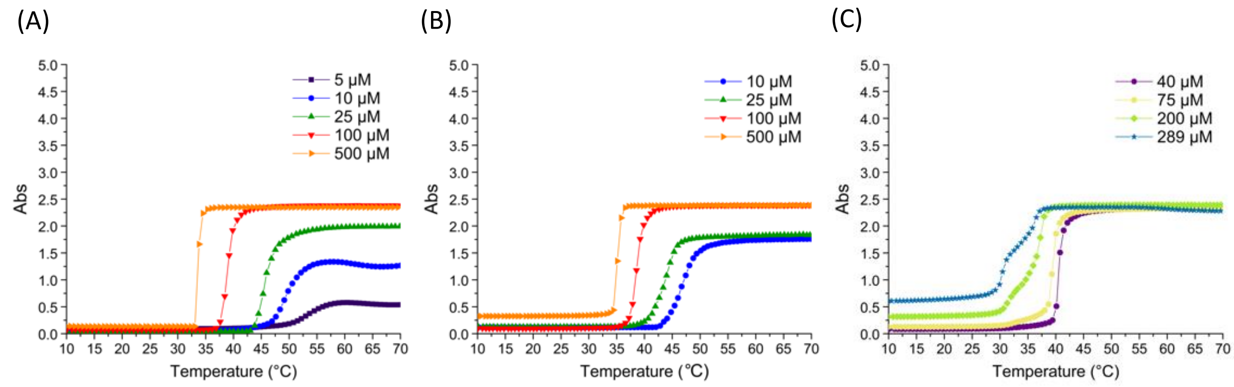

Figure S12. Turbidity assays at 350 nm with a rate of 1°C/min of (A, B, C) ELP-[*M$_1$V$_3$-*i*]-[I-*20*] (*i* = *20*, *40*, *60* respectively) in ultrapure water.

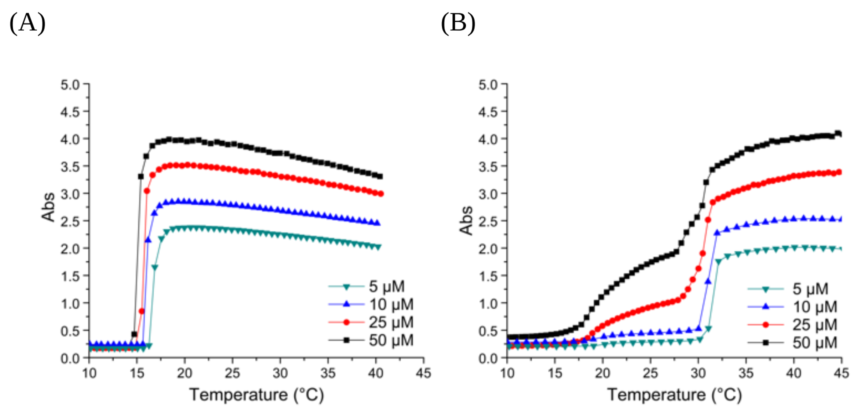

Figure S13. Turbidity assays at 350 nm with a rate of 1°C/min of (A) ELP-[M_1V_3-*60*]-[I-*90*] and (B) its oxidized counterpart, ELP-[*M_1V_3-*60*]-[I-*90*], in PBS.

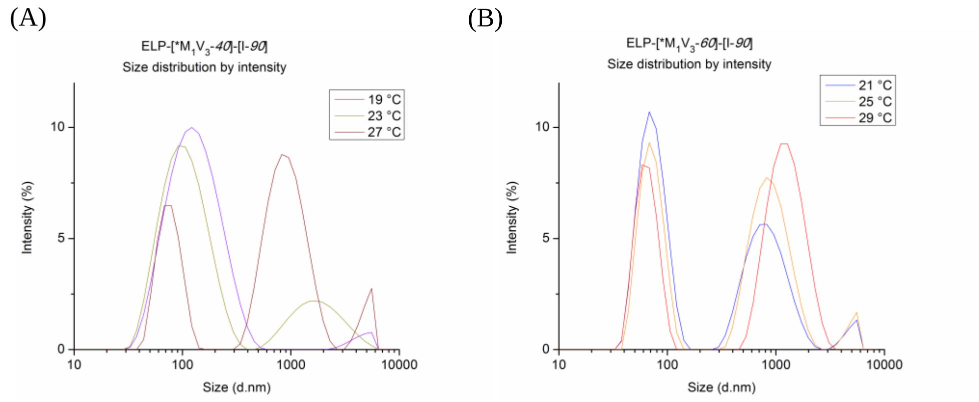

Figure S14. Size distribution by intensity of (A, B) ELP-[M_1V_3-*i*]- [I-*90*] (*i* = *40*, *60*, respectively) at 10 µM in PBS determined *via* DLS in the particle formation regime.
